# Antiviral activity of bovine type III interferon against bovine viral diarrhea virus is greatly reduced in bovine turbinate cells due to limited expression of IFN lambda receptor 1 (IL-28Rα)

**DOI:** 10.3389/fimmu.2024.1441908

**Published:** 2024-08-19

**Authors:** Rohana P. Dassanayake, Harish Menghwar, Kathryn A. Bickel, David J. Holthausen, Hao Ma, Fayna Diaz-San Segunda, Monica Rodriguez-Calzada, Gisselle N. Medina, Sarah Attreed, Shollie M. Falkenberg, Carly Kanipe, Randy E. Sacco, Teresa De Los Santos, Eduardo Casas

**Affiliations:** ^1^ Ruminant Diseases and Immunology Research Unit, Agricultural Research Service, National Animal Disease Center, United States Department of Agriculture, Ames, IA, United States; ^2^ ARS Research Participation Program, Oak Ridge Institute for Science and Education (ORISE), Oak Ridge, TN, United States; ^3^ Plum Island Animal Disease Center, North Atlantic Area, Agricultural Research Service, United States Department of Agriculture, Greenport, NY, United States; ^4^ National Bio and Agro-Defense Facility (NBAF), ARS, USDA, Manhattan, KS, United States; ^5^ Sugg Laboratory, Department of Pathobiology, College of Veterinary Medicine, Auburn University, Auburn, AL, United States; ^6^ Bacterial Diseases of Livestock Research Unit, National Animal Disease Center, Agricultural Research Service, USDA, Ames, IA, United States

**Keywords:** bovine turbinate primary epithelial cells, bovine type III interferon, bovine viral diarrhea virus, IFN-λ3, IL-28Rα, IL-10Rβ, Madin-Darby bovine kidney cells

## Abstract

**Introduction:**

The antiviral activity of recombinant bovine interferon lambda 3 (bovIFN-λ3) against bovine viral diarrhea virus (BVDV) has been demonstrated *in vitro* in Madin-Darby bovine kidney cells (MDBK) and *in vivo* in cattle. However, anti-BVDV activity of bovIFN-λ3 has not been studied in bovine respiratory tract epithelial cells, supposedly a primary target of BVDV infection when entering the host by the oronasal route.

**Methods:**

Here we investigated the anti-BVDV activity of bovIFN-λ3 in bovine turbinate-derived primary epithelial cells (BTu) using BVDV infection and immunoperoxidase staining, TCID_50_, RT-qPCR, DNA and transcriptome sequencing, and transfection with plasmids containing the two subunits, IL-28Rα and IL-10Rβ that constitute the bovIFN-λ3 receptor.

**Results:**

Our immunoperoxidase staining, RT-qPCR, and TCID_50_ results show that while BVDV was successfully cleared in MDBK cells treated with bovIFN-λ3 and bovIFN-α, only the latter, bovIFN-α, cleared BVDV in BTu cells. Preincubation of MDBK cells with bovIFN-λ3 before BVDV infection was needed to induce optimal antiviral state. Both cell types displayed intact type I and III IFN signaling pathways and expressed similar levels of IL-10Rβ subunit of the type III IFN receptor. Sequencing of PCR amplicon of the IL-28Rα subunit revealed intact transmembrane domain and lack of single nucleotide polymorphisms (SNPs) in BTu cells. However, RT-qPCR and transcriptomic analyses showed a lower expression of IL-28Rα transcripts in BTu cells as compared to MDBK cells. Interestingly, transfection of BTu cells with a plasmid encoding IL-28Rα subunit, but not IL-10Rβ subunit, established the bovIFN-λ3 sensitivity showing similar anti-BVDV activity to the response in MDBK cells.

**Conclusion:**

Our results demonstrate that the sensitivity of cells to bovIFN-λ3 depends not only on the quality but also of the quantity of the IL-28Rα subunit of the heterodimeric receptor. A reduction in IL-28Rα transcript expression was detected in BTu as compared to MDBK cells, despite the absence of spliced variants or SNPs. The establishment of bovIFN-λ3 induced anti-BVDV activity in BTu cells transfected with an IL-28Rα plasmid suggests that the level of expression of this receptor subunit is crucial for the specific antiviral activity of type III IFN in these cells.

## 1 Introduction

Bovine viral diarrhea virus (BVDV) is an enveloped, single-stranded, positive-sense RNA virus with an approximately 12.5 kb genome (1). It is a member of the genus *Pestivirus* (A: BVDV1 and B: BVDV2 species) and belongs to the *Flaviviviridae* family (2, 3). BVDV1 has 21 sub-genotypes (1a-1u) and BVDV2 has four sub-genotypes (2a-2d) (2, 4). Based on their unique *in vitro* cell culture characteristics, BVDV has two biotypes, cytopathic (cp) which induce cell death, and noncytopathic (ncp) (5). ncp BVDV is the predominant biotype isolated from animals. BVDV is primarily a pathogen of cattle causing acute and persistent infections (PI), but it can also affect other domestic and wild ruminant species (6, 7). Although BVDV PI calves may not develop signs of disease, they continuously shed BVDV and risk exposing other uninfected calves to BVDV infections that can lead to transient lymphocytopenia, lymph nodes and thymus lymphocytes depletion, and thus immunosuppression (8–10). Although acute BVDV infections are often subclinical, it can increase the risk for secondary bacterial and viral infections and is one of the viruses that can contribute to bovine respiratory disease complex (BRDC) that causes significant economic losses to the U.S. cattle industry (11–13).

In vertebrates, the first cellular response and defense mechanism against viral infection is the production of interferons (IFNs) (14–16). IFNs are a broad class of cytokines, and they are divided into three classes, type I, type II, and type III (17).Type I (IFN-α/β) and III (IFN-λs/interleukin (IL)-28/IL-29) IFNs have been identified as the most important mediators of innate immunity due to their antiviral activity and their ability to act as regulators for the development and activation of both, innate and adaptive immune responses. Once secreted, type I and type III IFNs act in an autocrine and paracrine manner (14–16). Despite type I and III IFNs binding to different receptors, they activate the same Janus-activated kinases (JAK1 and TYK2) and signal transducers and activators of transcription (STAT1 and STAT2), JAK-STAT signaling pathway, leading to the formation of IFN-stimulated gene factor 3 (ISGF3), a transcription complex which consists of STAT1, STAT2, and DNA-binding protein IFN regulatory factor 9 (IRF9) (15, 17–20). The ISGF3 complex (STAT1/STAT2/IRF9) translocates into the nucleus, binds to the IFN-stimulated response elements (ISRE) DNA sequences, an enhancer sequence present in the promoter of type I and III IFN-responsive genes, and then induces the expression of hundreds of IFN stimulated genes (ISGs) which mediate the IFNs biological effects (20).

It has been previously reported that *in vitro* infection of bovine cells with cp BVDV results in type I IFN (IFN-α/β) expression and cell death, whereas ncp BVDV fails to induce type I IFN expression and cell death (5, 21–24). The inhibition of type I IFN expression in cell culture by ncp BVDV is due to the inhibition of IFN regulatory factor 3 (IRF3) binding to DNA, after IRF3 translocated into the nucleus (25). Similarly, type I IFN expression differences have also been observed in *in vivo* early bovine fetal challenge studies, such that cp BVDV but not ncp BVDV (6). In contrast, acute infection of postnatal naïve calves with ncp BVDV resulted in type I IFN production (26, 27). The leukocyte subset that was found to produce type I IFNs in lymph nodes was positive for myeloid markers CD14, CD11b, and CD172a, but negative for CD4 and CD45RB (26).

Based on sequence conservation, type I IFNs classified as -α (13 subtypes in humans) and a single member of each of -β, -ϵ, -κ, -ω, -δ, -ζ, -τ, -δ and, -ν whereas, type II IFNs has only one member, IFN-γ (28–30). Type III IFN, which is known as IFN-λ, has been identified in several species including cattle (15, 16, 31). In the type III IFN family, four structurally related members have been identified in humans, IFN-λ1 (IL-29), IFN-λ2 (IL28A), IFN-λ3 (IL-28B), and IFN-λ4 (p179) that are distinctly related to type I IFNs and the IL-10 family (15, 16, 18, 32). More recently, a gene encoding IFN-λ3 has been identified from embryonic bovine kidney cells (31, 33). Type I IFNs are produced by most cell types, while type II IFN is predominantly produced by natural killer cells and T lymphocytes, although myeloid cells (macrophages and dendritic cells) can also produce type II IFN (34, 35). Despite epithelial cells being the major production site of type III IFNs, macrophages, monocytes, and dendritic cells can also produce type III IFNs (35, 36). Type I IFNs receptors (IFNAR1 and IFNAR2) and type II receptors (IFNGR1 and IFNGR2) are expressed in most nucleated cells and therefore, their function is systemic (30, 37). In contrast, type III IFN receptor complex is composed of a widely expressed and shared IL-10R2 (IL-10Rβ) subunit and a unique IFN-λR1 (IL-28Rα) subunit which is responsible for signal transduction for this family of IFNs (15, 16, 20). This unique IL-28Rα expression in mice and humans is largely restricted to the cells of epithelial origin and the liver, and thus might reduce or prevent virus entry through skin and mucosal surfaces (38–41).

The antiviral activity of bovine IFN-λ3 against several viruses including BVDV has previously been characterized *in vitro* using bovine kidney cells (embryonic bovine kidney and Madin-Darby bovine kidney cells [MDBK]) and *in vivo* in cattle (31, 33, 42, 43). It has been previously shown that following intranasal BVDV entry, virus first replicates in the nasal mucosa (turbinates) and to high titers in the tonsil before spreading the virus to the other lymph nodes and then white blood cells disseminates virus to visceral organs (44, 45). Although a typical BVDV entry site in cattle is the mucosal epithelium in respiratory and digestive tracts, effects of bovine IFN-λ3 on primary respiratory or digestive tract mucosal epithelial cells have not been reported previously. Air passes to the lungs through the nasal meatus lined by turbinates, which are small structures found inside the nose/nostrils. Turbinates, in turn, are lined with epithelial cells. Rostrally, there are squamous epithelial cells. In the mid and posterior areas, turbinates are lined by pseudostratified ciliated epithelial cells, some goblet cells, and non-ciliated sensory cells. The most important function of nasal epithelial cells is to serve as a physical barrier and cilia transit of particulate material. However, these cells also produce various innate immune products, chemokines, and cytokines which control innate and adaptive immune systems. We and others have previously demonstrated that BVDV replicates efficiently in bovine turbinate epithelial cells and MDBK cells reaching similar end-point viral titers in the two cell types (46–48). Therefore, the goal of this study was to characterize the antiviral activity of bovine IFN-λ3 against BVDV in primary target bovine nasal turbinate-derived primary respiratory mucosal epithelial (BTu) cells.

## 2 Materials and methods

### 2.1 Cells and virus

Madin-Darby bovine kidney cell line (MDBK, CCL-22) and primary neonatal bovine turbinate cells (BTu, CRL-1390) were obtained from the American Type Culture Collection (ATCC, Manassas, VA). Four additional low-passaged primary neonatal BTu cells (BTu-10/07; BTu-9/08, BTu-12/14, and BoTur, under 10 passage) prepared from four animals were also included in the study. BoTur cells were kindly provided by the National Veterinary Services Laboratories, Animal Plant and Health Inspection Service (NVSL, APHIS, Ames, IA). One ncp bovine viral diarrhea virus (BVDV) strain AU-PI-28 (PI28, sub-genotype BVDV-2a) from a persistently infected cow was used in this study (49, 50). PI28 stocks were prepared from infected MDBK cells (50, 51) and titrated (tissue culture infection dose 50/mL, TCID_50_) using an immunoperoxidase staining method (52). The Reed-Muench method was used to calculate TCID_50_.

### 2.2 Antibodies and reagents

Recombinant bovine IFN-λ3 rich supernatants (bovIFN-λ3) were harvested from Mike and Gisselle Porcine Kidney cells (MGPKavB6) infected with an Ad5-bovIFN-λ3 vector, 24 hours post-infection, following the method previously described (31, 42, 53). Recombinant bovine IFN-α (bovIFN-α) was purchased from KingFisher Biotech (Minneapolis, MN). 96-well clear flat bottom tissue-culture treated microplate was purchased from Costar (Corning, NY). 96-well optical bottom (polymer coverslip) μ-plates were purchased from Ibidi USA, Inc (Fitchburg, WI). Monoclonal antibody (mAb, clone N2) raised against BVDV E2 structural protein was previously produced in our laboratory. Minimal Essential Medium (MEM), L-Glutamine, recombinant protein G-HRP conjugate, VetMAX™-Gold BVDV PI Detection Kits, Qubit broad range RNA assay kits, pcDNA3.4 TOPO TA mammalian expression vector, lipofectamine 3000 transfection reagent, Superscript III reverse transcriptase, and oligo(dT)_20_ primer were purchased from ThermoFisher Scientific (Grand Island, NY). Goat anti-mouse (IgG) antibody was purchased from MP Biomedicals (Santa Ana, CA). Fetal bovine serum (FBS) was purchased from PAA Laboratories Inc (Ontario, Canada). Anti-FLAG M2 monoclonal antibody (IgG1), Tween 20, antibiotic-antimycotic, and 3-amino-9-ethylcarbazole (AEC) were purchased from Sigma (St. Louis, MO). Alexa Fluor 488 conjugated rat anti-mouse IgG1 was purchased from Biolegend (San Diego, CA). Taq Hot Start Quick-Load 2× Master Mix with GC buffer, Luna Universal One-Step RT-qPCR Kit (master mix), NEBNext rRNA Depletion Kit (Human/Mouse/Rat), NEBNext Ultra II RNA Library prep kits were purchased from New England Biolabs (Ipswich, MA). Codon-optimized bovine type III interferon receptors IL-28Rα (with or without N-terminus FLAG epitope; GenBank Accession no. XM_868941.2) and IL-10Rβ (GenBank Accession no. NM_001076975) genes (gBlock gene fragments), primers, and FAM-labeled *Taq*Man minor groove binding (MGB) probes were synthesized by Integrated DNA Technologies (Coralville, IA). Codon-optimized bovine Mx1 (Myxovirus resistance; GenBank Accession no. NM_173940.2) gene cloned into a mammalian expression pcDNA3.1/Hygro(+) vector was purchased from Genscript (Piscataway, NJ). Total cellular RNA extraction mini kits and viral RNA extract kits were purchased from Qiagen (Valencia, CA).

### 2.3 Cell culture

Both MDBK and BTu cells were maintained in MEM supplemented with 10% (v/v) filtered and heat-inactivated FBS (HI-FBS), L-Glutamine, antibiotic-antimycotic, and incubated at 37°C in a humidified atmosphere of 5% CO_2_. The FBS, BTu, and MDBK cells were confirmed free of pestivirus RNA and antibodies.

### 2.4 Epithelial cells and bovIFN-λ3 antiviral activity

Antiviral activity of bovIFN-λ3 and bovIFN-α against BVDV-2a (PI28) was initially tested *in vitro* in MDBK cells as described previously, but with some modifications (31, 42, 54). First, an initial experiment was set to identify the multiplicity of infection (MOI), the optimal frequency of IFNs treatments, and their respective concentrations. Briefly, 50 µL of MDBK cells suspended in complete MEM (cMEM, 2 × 10^5^ cells/mL) were transferred into each well of a Costar 96-well clear flat bottom tissue-culture treated microplate or ibidi 96-well optical bottom (polymer coverslip) μ-plates and allowed to adhere for about 2 hours. 50 µL of serially two-fold diluted bovIFN-λ3 (1:4 - 1:128) or bovIFN-α (100 - 3.125 ng/mL) were added (two replicates per each dilution, day -1) to the cells, followed by a 24 hour incubation at 37°C and 5% CO_2_. Cells were then incubated with BVDV-2a (PI28) at MOI of 0.5, 0.05, and 0.005 for 1 hour, after which cells were washed twice with MEM. The IFN treatment was administered under two different conditions (1). 100 µL of fresh cMEM without any IFNs added (IFNs only on day -1) and (2) 50 µL of diluted IFNs were added daily for three consecutive days (day 0, day 1 and day 2) post-BVDV infection. Cells incubated with IFNs (one or four days in different combinations without BVDV), BVDV only (no IFNs treatment), and cells without any treatment (no IFNs or BVDV) were used as controls. On day four post (first) IFNs treatment (or day 3 post-BVDV infection), amount of virus was measured by immunoperoxidase staining and RT-qPCR (VetMAX™-Gold BVDV PI Detection Kit), as described in sections 2.5 and 2.6.

After determining the optimal BVDV MOI (0.5) and the most effective IFN treatment duration (one day vs four days), subsequent experiments were conducted to identify the optimal frequency of bovIFN-λ3 treatment for detection of maximum antiviral activity. MDBK cells were plated and treated with serially diluted bovIFN-λ3 (1:4 – 1:128) on day -1 as described previously. The following day, cells were infected with BVDV (PI28) at MOI of 0.5 for 1 hour and then washed. Different treatments (final volume = 100 µL/well) were added into each well (day 0) such as [i] one-time bovIFN-λ3 treatment (24 hours before BVDV infection; day -1 only), [ii] bovIFN-λ3 were added 2-3 days in different combinations (day -1 and day 0; day -1 and day 1; day -1 and day 2; day -1, day 0 and day 1, day 0, day and day 2), and [iii] bovIFN-λ3 were added every day for 4 consecutive days [day -1, day 0, day 1, and day 2). In another experiment, MDBK cells were incubated simultaneously with bovIFN-λ3 and BVDV (MOI 0.5) on day 0 and followed 2-3 different days of bovIFN-λ3 treatment as described previously.

Since bovIFN-λ3 treatment of MDBK cells at day -1/day 0 and day -1/day 1 were very similar to four consecutive bovIFN-λ3 treatment in-terms of maximum antiviral activity against BVDV, only these two treatment combinations were employed in subsequent experiments involving BVDV-infected BTu cells across five different BTu cell preparations. BovIFN-λ3 similarly treated MDBK cells were used as an assay control.

Likewise, after determining optimal bovIFN-α, treatment (one day *vs* four days), next sets of experiments were conducted to identify frequency of bovIFN-α treatment to define optimal antiviral activity in BTu cells. BTu cells were incubated with serially diluted bovIFN-α (100 - 3.14 ng/mL) on day -1 as described previously. The following day, cells were infected with BVDV (PI28) at MOI of 0.5 for 1 hour and washed. As described for MDBK cells, different treatments (final volume = 100 µL/well) were added into each well (day 0) such as [i] bovIFN-αwere added 2-3 days in different combinations, and [ii] bovIFN-α were added every day for 4 consecutive days. BTu cells were prepared for immunoperoxidase staining to detect BVDV infected cells as described in section 2.5.

### 2.5 BVDV immunoperoxidase staining

BVDV presence was determined using immunoperoxidase staining as described previously, but with minor modifications (52, 55). The cells were fixed in 40% acetone solution in phosphate-buffered saline (PBS)/bovine serum albumin (0.02% w/v) for 10 minutes at room temperature (RT) and air dried at 37°C. 50 µL of diluted anti-BVDV N2 mAb (3, 56) in PBSTN (0.01% Tween 20, 2.95% sodium chloride) buffer was added and plates were incubated at RT for 1 hour. Plates were washed with PBST (0.05% Tween 20) and incubated with goat anti-mouse antibody (IgG) at RT for 1 hour. After washing with PBST, recombinant protein G-HRP conjugate (horseradish peroxidase) was added and incubated at RT for 1 hour followed by washing with PBST. Plates were incubated with HRP substrate, AEC in hydrogen peroxide/acetate buffer (1:10 v/v) at RT for about 10 minutes, decant AEC, and wells were filled with (tap) water. BVDV staining, appearing as red/brown only in the cytoplasm of infected cells (optical bottom ibidi µ plates), was visualized using a Leica DM IL LED inverted microscope and images were captured using a Leica MC120 HD camera (Leica Microsystems Inc., Buffalo Grove, IL). Images were obtained with Leica High Plan CY 10× objective lens at numerical aperture 0.25. Final figures were prepared using Adobe Photoshop Elements 11.

### 2.6 Quantification of BVDV RNA

BVDV viral loads in BTu and MDBK cells with or without bovIFN-λ3 treatment were quantified using a commercially available VetMAX™-Gold BVDV PI Detection Kit as described by the manufacturer. BVDV RNA was extracted using QIAcube RNA/DNA extraction instrument and quantified using Qubit broad range RNA assay kit. The relative quantities of BVDV in each sample were calculated using a standard curve generated from the serial dilution of RNA from a known TCID_50_ quantity of BVDV (PI28). Bar charts were prepared using Microsoft Excel software and final figures were prepared using Adobe Photoshop Elements 11.

### 2.7 Cloning of bovine IL-28Rα, IL-10Rβ, and Mx1 genes

IL-28Rα and IL-10Rβ gBlock gene fragments were synthesized and individually cloned into a mammalian expression vector (pcDNA3.4 TOPO TA). Bovine Mx1 gene was synthesized and cloned into a mammalian expression pcDNA3.1/Hygro(+) vector.

### 2.8 BTu cells and plasmid transfection

50 µL of BTu cells in cMEM medium (2 × 10^5^ cells/mL) were transferred to each well in a 96-well plate. Approximately 2 hours later, BTu cells were transiently transfected with plasmids (individual pcDNA3.4/IL-28Rα, pcDNA3.4/IL-10Rβ, pcDNA3.1/Mx1 or both pcDNA3.4/IL-28Rα and pcDNA3.4/IL-10Rβ plasmids, 10 replicates each) using lipofectamine 3000 transfection reagent. Two hours post-transfection, 50 µL of diluted bovIFN-λ3 (only at 1:16 dilution, day -1) was added into appropriate wells and incubate for 24 hours at 37°C and 5% CO_2_. Twenty-four hours post-transfection, BTu cells were infected with BVDV-2a (PI28, MOI 0.5) as described in section 2.4. After washing, bovIFN-λ3 (at 1:16 dilution) was added to the appropriate wells on day 0, day 1, and day 2 (4 consecutive days). On day 4 post-bovIFN-λ3 treatment, BTu cells were prepared for BVDV immunoperoxidase staining.

### 2.9 Flow cytometry

IL-28Rα expression plasmid was constructed by placing FLAG epitope at the end of signal sequence (between 20-21 amino acids) and BTu cells in 6-well plate (~7 × 10^5^ cells/well) were transfected using lipofectamine 3000. Approximately 24 hours post-transfection, cells were detached using 0.25 mM EDTA/PBS pH 7.4, washed, and incubated with anti-FLAG mAb (IgG1) at RT for 15 minutes. BTu cells were washed twice with stain buffer and incubated with AF 488 anti-mouse IgG1 mAb at RT for 15 minutes. Cells were washed twice with stain buffer and resuspended in stabilizing fixative. Similarly treated un-transfected BTu cells were used as a negative control. Flow cytometric analysis was performed using a BD FACSymphony™ A3 flow cytometer (BD Biosciences). AF 488 was excited using 488 nm laser and emission signal was collected with 515/20 nm band-pass filter. Cells were visualized in forward and side light scatter and electronic gates were placed to contain cells at single cell levels. Approximately 10,000 events were collected for each sample for data analysis.

### 2.10 RT-qPCR and gene expression analysis

BTu and MDBK cells (~2 × 10^6^ cells) were seeded into 25 cm^2^ flasks as described previously. Two hours post-plating, cells incubated with BVDV at MOI of 0.5, bovIFN-α (100 ng/mL), bovIFN-λ3 (1:16 dilution), or kept uninfected/untreated (control). Twenty four hours post-treatments, total cellular RNA was extracted using RNeasy Mini Kit. Purified RNA was quantified using Qubit broad range RNA assay kit, aliquoted, and stored at -20°C until used. Primers and probes used in reverse transcription quantitative real-time polymerase chain reaction (RT-qPCR) assay are shown in **Table 1**. Several primers and *Taq*Man MGB probes used in this study were designed previously (31). When new primers and probes were needed, IDT PrimerQuest Tool was used. Bovine glyceraldehyde 3-phosphate dehydrogenase (GAPDH) was used as housekeeping gene and to normalize PCR results. A one-step quantitative RT-qPCR assay was performed using Luna Universal One-Step RT-qPCR Kit (master mix) as described by the manufacturer on an Applied Biosystems Quant Studio 5 Real-Time PCR System. Briefly, one-step RT-qPCR amplification was carried out in 20 µL reaction volume with the following cycling conditions: 55°C for 10 minutes (1 cycle, reverse transcription), 95°C for 1 minute (1 cycle, denaturation), followed by 40 cycles of 95°C for 10 seconds (denaturation), and 60°C for 30 seconds (annealing and extension). At least two technical replicates were used to obtain each average Ct value.

**Table 1:** Bovine oligonucleotide primers and probes used for real-time RT-qPCR.

Gene	Accession no.	Forward primer (5`-3`)	TaqMan probe (5`-3`) (5`6-FAM ZEN-3`//31Iowa Black FG	Reverse primer (5`-3`)
JAK1	NM_001206534	GGACCAACGACAATGAACAATC	AGATGCAACACCTCTCCTTGACGC	TGTCCCTGAGCAAACAGATAC
STAT1	NM_001077900	TTCAGGAAGACCCAATACAGATG	CCTGGCTGATTCTCTGGGCATGAT	CAAGCTCCTTCTGTTTGTCTAAC
STAT2	NM_001205689	CCTTGAGCCCTTCTGACTAC	ATTTGCCCCTCGTACCCCTCAC	CCCCAGAGTTAACCCAGTTTAG
IRF7	BC151518	GGACTGTGACACGCCCATCT*	ACTTCGGCACCTTCT*	CCCGGAACTCCAGCAGTTC*
IRF9	NM_001024506	CAGTTCTGCATCCTCTGAGAAG	ACTGCATACTCAGCCCCTCGTTG	TGCTCCCAAAGTCTAAACGG
IFN-λ3	HQ317919	ACTCATCCCTGGGCCACA*	CCTGGAGCAGCCCCTTCTCACG*	GCTTGGAGTGGATGTTCTGCA*
MX1	AF047692.1	AGACGAGTGGAAAGGCAAAG	TGCTTCACAGGTGGAAAAGGAAATCAGT	CCTCCAGACTAATCAGCTCATG
GAPDH	NM_001034034	GCATCGTGGAGGGACTTATGA*	CACTGTCCACGCCATCACTGCCA*	GGGCCATCCACAGTCTTCTG*
IFNAR1	NM_174552	ACATGGTATGAGGTTGAGCC	CCCCAGATGTGCATTTAGAAGCTGAAGA	AACGATCCATAGCCCACATG
IFNAR2	NM_174553	GTAATGATACTGAAACGGATTGGC	TCGGCAAACACCCAGACTGACA	TGAATGACTTCCACGGTAGC
IL10-Rβ	NM 001076975	TTTGACAAACTGAGCGTCATCA*	AAGTGTCTGAAAGCTGCAA*	CGGCCCCAGGGTTCA*
IL28-Rα	XM_868941.2	CTGCACGACTTGATCGTATGT	CAGCCTGACCCTTCTGGTCACTTC	TCCTCAGTGCTGTCCTTCT
	TM (Exons 5 & 6)	GAGCCCACCTGCTTCTTC	ATTTCTGCTTCCACTGCTGTTGGC	CCTGAAGGTCTTCCACATCAC
	Exon 3	GAATGTGACGCTGCTCTC	CTTCAGTGTGTACCTGACGTGGCT	AAAGCTTTGATAGGCCACA

*Primer sequences were adopted from Diaz-San Segunda et al. (2011). TM, transmembrane domain.

The expression levels of both type I and type III IFN receptors (IFNAR1/IFNAR2 and IL-28Rα/IL10-Rβ, respectively) were assessed in bovIFN-α and bovIFN-λ3 treated, BVDV infected and uninfected MDBK and BTu cells. In addition to exon 7 specific primers, transmembrane-specific (TM) primers were also tested for IL-28Rα subunit (**Table 1**). Relative quantities (RQ) of type I and III IFN signaling pathway genes, IFN receptor genes expression levels, and Mx1 (mRNA transcripts) were calculated using [RQ = 2(−ΔΔCt)] method. 2^(-ΔΔCt)^ where ΔCt = AvgCt of GOI − AvgCt of GAPDH, and ΔΔCt = ΔCt of BVDV infected or IFN treated cells − ΔCt of control cells (31, 57).

### 2.11 Cloning and sequencing of IL-28Rα

Total cellular RNA extracted from bovIFN-λ3 treated MDBK and BTu cells were used for cDNA synthesis using Superscript III reverse transcriptase and oligo(dT)_20_ primer as described by the manufacturer. IL-28Rα gene was PCR amplified with untranslated region (UTR, locations are shown in the primer) specific forward primer (IL-28Rα-F7) 5`-GTGAACCAGGAGGCCAGA-3`, or (IL-28Rα-F17) 5`-AGGCCAGAGGAGTGGGA-3` and reverse primer (IL-28Rα-R2142) 5`- CCACCCTAGGGAACTCATAAAC using One*Taq* Hot Start Quick-Load 2× Master Mix with GC buffer. PCR amplification was carried out in 25-50 µL reaction volume with the following cycling conditions: 94°C for 2 minutes (1 cycle), 35 cycles of 94°C for 30 seconds (denaturation), 52°C for 30 seconds (annealing), and 68°C for 2 minutes (extension) with a final extension at 68°C for 5 minutes. PCR products purified from agarose gel were directly sequenced. In addition to full-length IL-28Rα subunit PCR products, exons 2-3, 5-6 and 7 specific PCR products obtained from RT-qPCR product were also sequenced.

### 2.12 Transcriptome analysis using RNA-Seq

Total cellular RNA was extracted from BTu and MDBK cells using RNeasy Mini Kit. The RNA samples with RNA Integrity Number (RIN) of 9.5 and 10 (triplicate samples per each cell type) were selected for library preparation. Ribosomal RNA (rRNA) was removed using NEBNext rRNA Depletion Kit (Human/Mouse/Rat) and transcriptomic libraries (three per each cell type) were prepared using NEBNext Ultra II RNA Library prep kit for Illumina. The libraries were sequenced by Illumina NovaSeq 6000 Sequencing System with 2 × 150 bp paired ends. Library preparation and sequencing were performed at the Iowa State University DNA Facility (Ames, IA). The quality of the transcriptomic library raw reads was evaluated using FastQC (58). Adapters and low-quality reads (<Q25) were removed with Cutadapt (version 4.0). The cleaned reads for BTu cells ranged from 18,273,076 to 24,233,100 and for MDBK cells ranged from 9,855,258 to 23,879,634. Normalized reads were obtained by using STAR (2.7.10b) and RSEM (1.3.3), and Bos taurus genome resources in Ensembl (version 106) as reference (59). Burrow-Wheeler Aligner (BWA, version 0.7.17) was used to align the cleaned reads to reference transcript. The SAM files were converted to BAM files with samtools (version 1.17) and visualized in Integrative Genomics Viewer (http://www.broadinstitute.org/igv) (60, 61).

## 3 Results

### 3.1 bovIFN-λ3 induces antiviral activity against BVDV in MDBK cells

**Figure 1:**
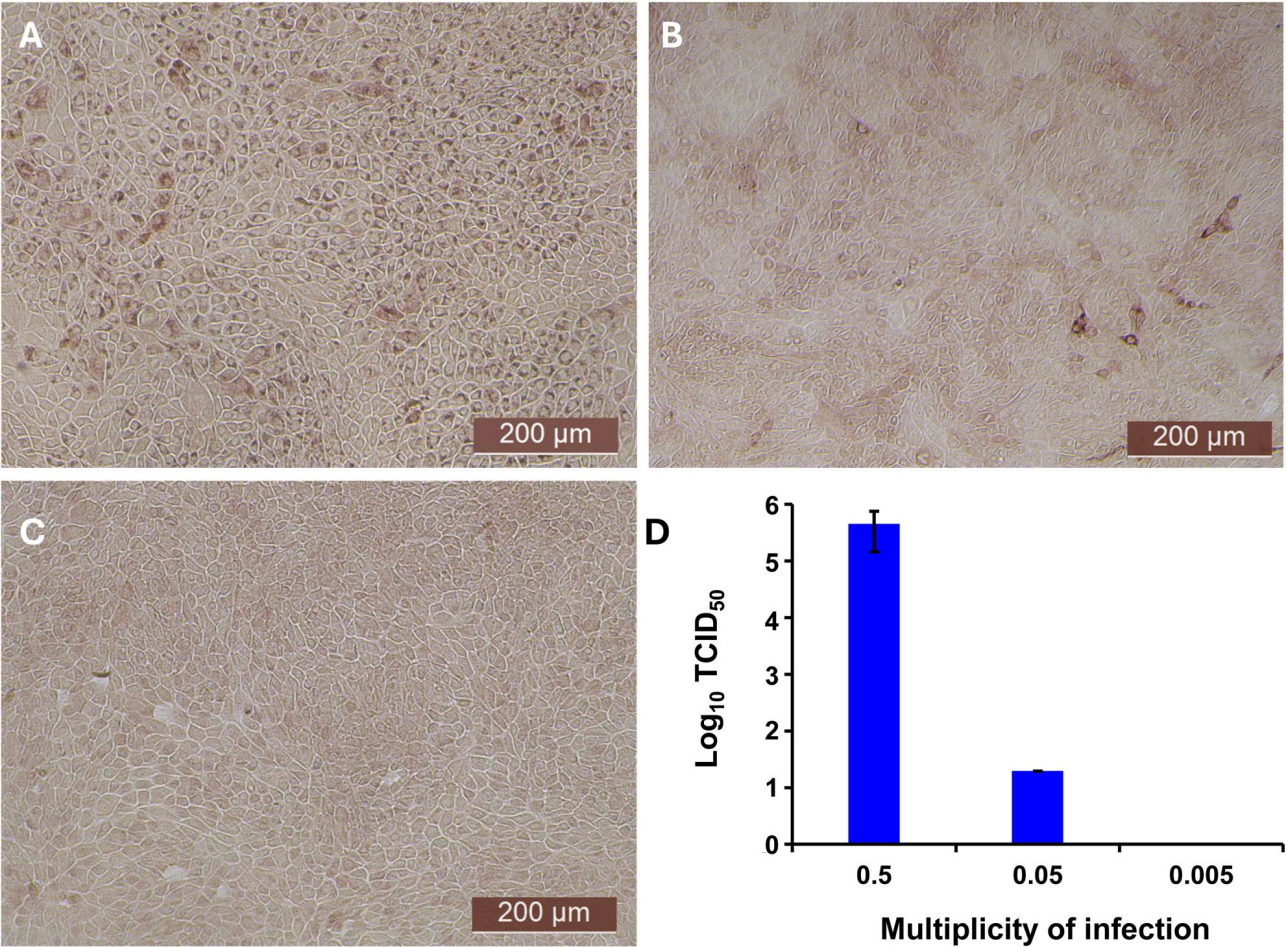
Assessment of ncp BVDV infection status in cultured Madin-Darby bovine kidney cell line (MDBK) by immunoperoxidase staining and RT-qPCR methods. **(A)** MDBK cells infected with BVDV (PI28) at multiplicity of infection (MOI) of 0.5. **(B)** MOI of 0.05. **(C)** MOI of 0.005. **(D)** BVDV titers in each sample after 3 days post-incubation is indicated as TCID_50_. BVDV was identified by immunoperoxidase staining using anti-E2 protein specific monoclonal N2 antibody. BVDV staining (red/brown) is restricted to the cytoplasm. Scale bar = 200 µm. BVDV titers were determined by BVDV RT-qPCR assay (VetMAX™-Gold BVDV PI Detection Kit) and expressed as TCID_50_. Statistical significance was determined by one-way ANOVA (*P<0.5).

The antiviral activity of bovIFN-λ3 has been previously reported in MDBK cells under particular assay conditions. These included pre-incubating cells in 6-well plates with bovIFN-λ3 for 24 hours (single treatment), followed by infection with BVDV, Vesicular Stomatitis Virus (VSV), or Foot-and-Mouth Disease Virus (FMDV) in at 100 focus forming units/well or at MOI of 1, overlaying with low-melting agarose (or gum tragacanth), and incubating for 24-48 hours to detect viral plaques by staining with crystal violet (31, 42). However, since an alternative method involving 96-well plates and detection of the BVDV E2 protein with a specific monoclonal antibody and immunoperoxidase staining is more commonly used for determining BVDV, we decided to apply it in our studies to measure sensitivity to IFNs. To identify optimal BVDV titer(s) for IFN testing, MDBK cells were incubated with BVDV at MOI of 0.5, 0.05, and 0.005 for 3 days followed by immunoperoxidase staining of BVDV E2 protein. Consistently, >90% of BVDV-infected (or positive) cells were found only at an MOI of 0.5 (**Figure 1A**) which was subsequently used for further experiments. Except for one well (MOI 0.05, **Figure 1B**), no BVDV positive cells were detected at MOI 0.05 or 0.005 (**Figure 1C**). Similar to BVDV immunoperoxidase staining findings, higher BVDV titers were observed with cells infected with BVDV at MOI of 0.5 (TCID_50_ = ~4.5 × 10^5^) compared to MOI of 0.05 (TCID_50_ = ~20) or 0.005 (TCID_50_ = 0) (**Figure 1D**).

**Figure 2:**
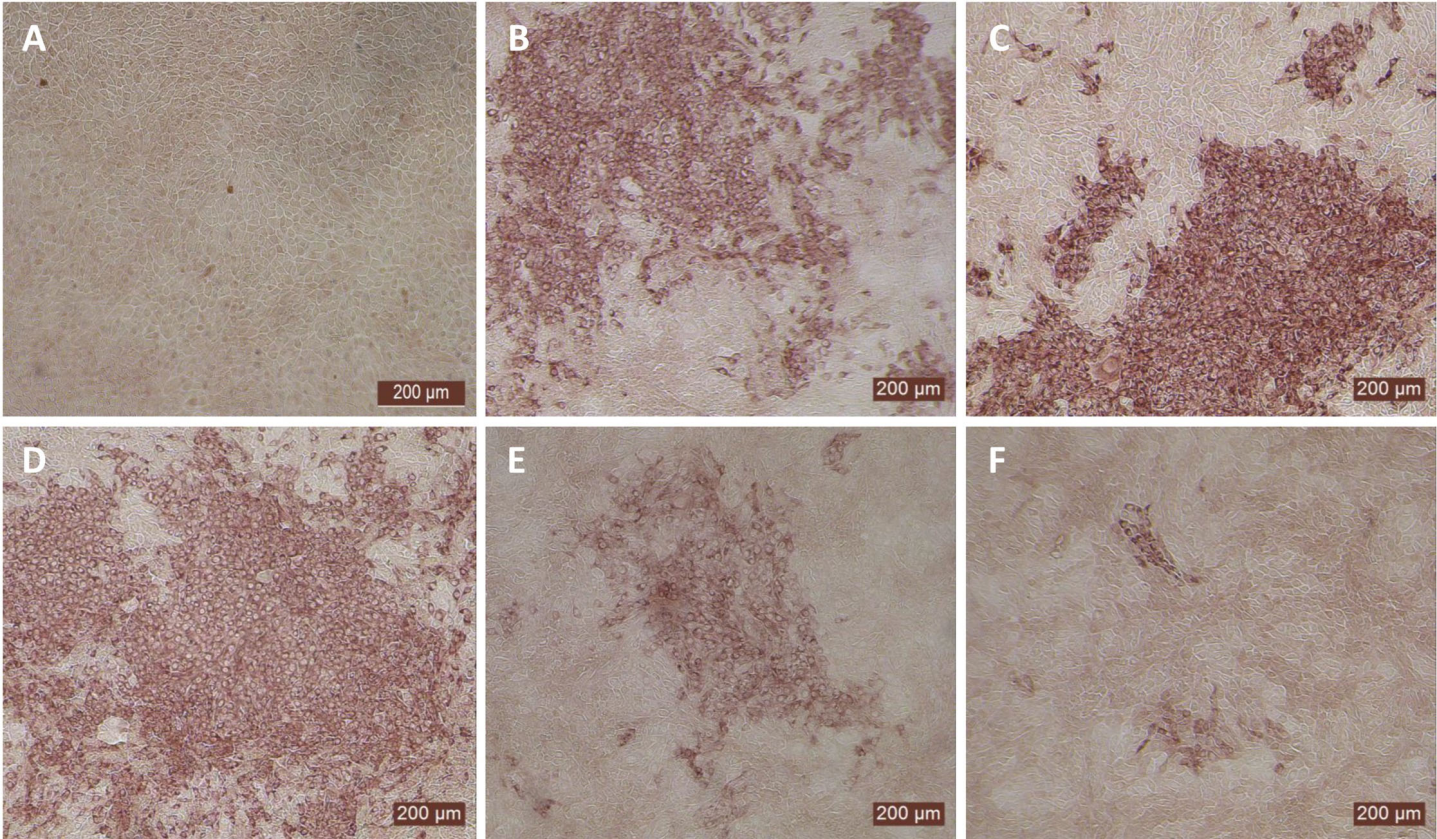
Antiviral activity of bovIFN-α and bovIFN-λ3 against ncp BVDV in cultured Madin-Darby bovine kidney cell line (MDBK). **(A)** Uninfected MDBK cells. **(B)** MDBK cells treated with bovIFN-α (100 ng/mL) 24 hours before BVDV infection (day -1 only). **(C)** MDBK cells treated with bovIFN-λ3 (1:16 dilution) 24 hours before BVDV infection (day -1 only). **(D)** BVDV infected MDBK cells (no IFN treatment). **(E)** MDBK cells treated with bovIFN-α (100 ng/mL) 24 hours before BVDV infection (day -1) followed by three additional days of bovIFN-α treatment (day 0, day 1 and day 2). **(F)** MDBK cells treated with bovIFN-λ3 (1:16 dilution) 24 hours before BVDV infection (day -1) followed by three additional days of bovIFN-λ3 treatment (day 0, day 1 and day 2). ncp BVDV2a PI28 strain at MOI of 0.5 was used to infect MDBK cells. BVDV was identified by immunoperoxidase staining using anti-E2 protein specific monoclonal N2 antibody. BVDV staining (red/brown) is restricted to the cytoplasm.

**Figure 3:**
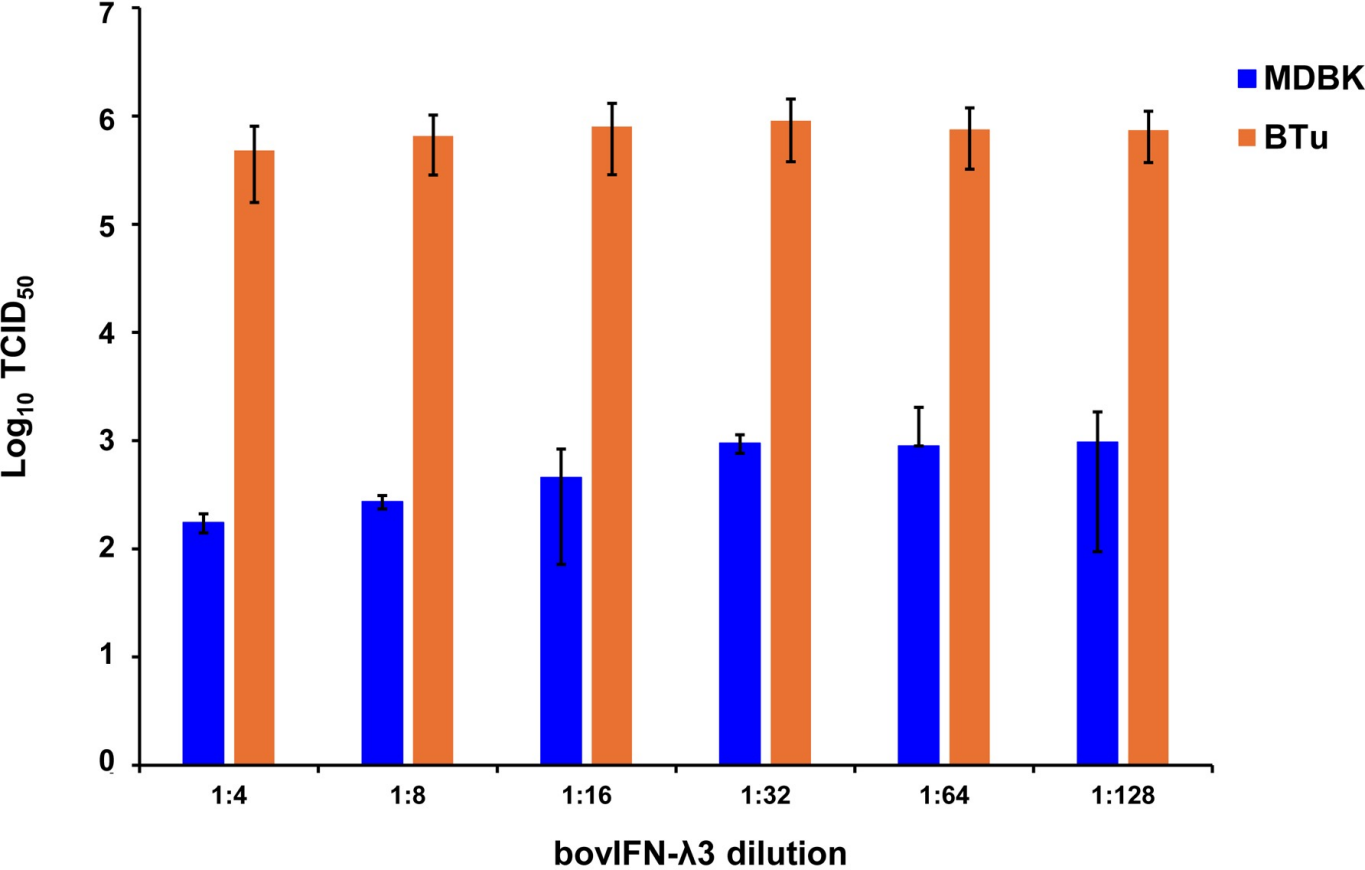
Antiviral activity of bovIFN-λ3 against ncp BVDV in cultured primary bovine turbinate epithelial cells (BTu) and Madin-Darby bovine kidney cell line (MDBK). BTu and MDBK cells were preincubated with bovIFN-λ3 24 hours before BVDV infection (day -1) followed by three additional days of treatments (day 0, day 1 and day 2). BVDV titers were determined by BVDV RT-qPCR (VetMAX™-Gold BVDV PI Detection Kit) assay and expressed as TCID_50_.

After determining the optimal BVDV titers (MOI 0.5), our next goal was to identify whether MDBK cells treated once (at day -1) or on four consecutive days with bovIFN-λ3 or bovIFN-α were needed for virus clearance. MDBK cells that received four consecutive days of bovIFN-λ3 treatment showed the greatest reduction in BVDV staining, with only a few positive cells (**Figure 2F**). In contrast, MDBK cells treated once on day -1 with bovIFN-λ3 showed approximately 60% BVDV positive cells irrespective of the IFN concentrations tested (**Figure 2C**). Similarly, when MDBK cells were treated once (day -1) with bovIFN-α, approximately 80% of the cells were BVDV-positive even at the highest IFN tested concentrations (100 ng/mL; **Figure 2B**). However, a greater reduction in BVDV staining (~20% BVDV positive cells, **Figure 2E**) was found when cells were treated with bovIFN-α for four consecutive days, particularly at higher bovIFN-α concentrations (50-100 ng/mL). In contrast, more than 50% cells were positive for BVDV staining at low bovIFN-α concentrations (3.2-25 ng/mL). Regardless of whether MDBK cells were treated once or for four days, bovIFN-λ3 showed a stronger antiviral activity against BVDV than bovIFN-α (**Figure 2**). As expected, MDBK cells in control wells showed no BVDV staining (**Figure 2A**) while infected wells showed over 90% staining (**Figure 2D**). It is important to highlight that BVDV transcription and replication is restricted to the cytosol (and not at the nuclear level) and therefore, BVDV immunoperoxidase staining is visible only in the cytoplasm. Similar to results obtained by BVDV immunoperoxidase staining, MDBK cells infected with BVDV at MOI of 0.5 and treated for four consecutive days with varying dilutions of bovIFN-λ3 had lower viral titers (TCID_50_ = ~175 to 1,000) (**Figure 3**) compared to untreated BVDV infected cells (TCID_50_ = ~4.5 × 10^5^) (**Figure 1D**).

**Figure 4:**
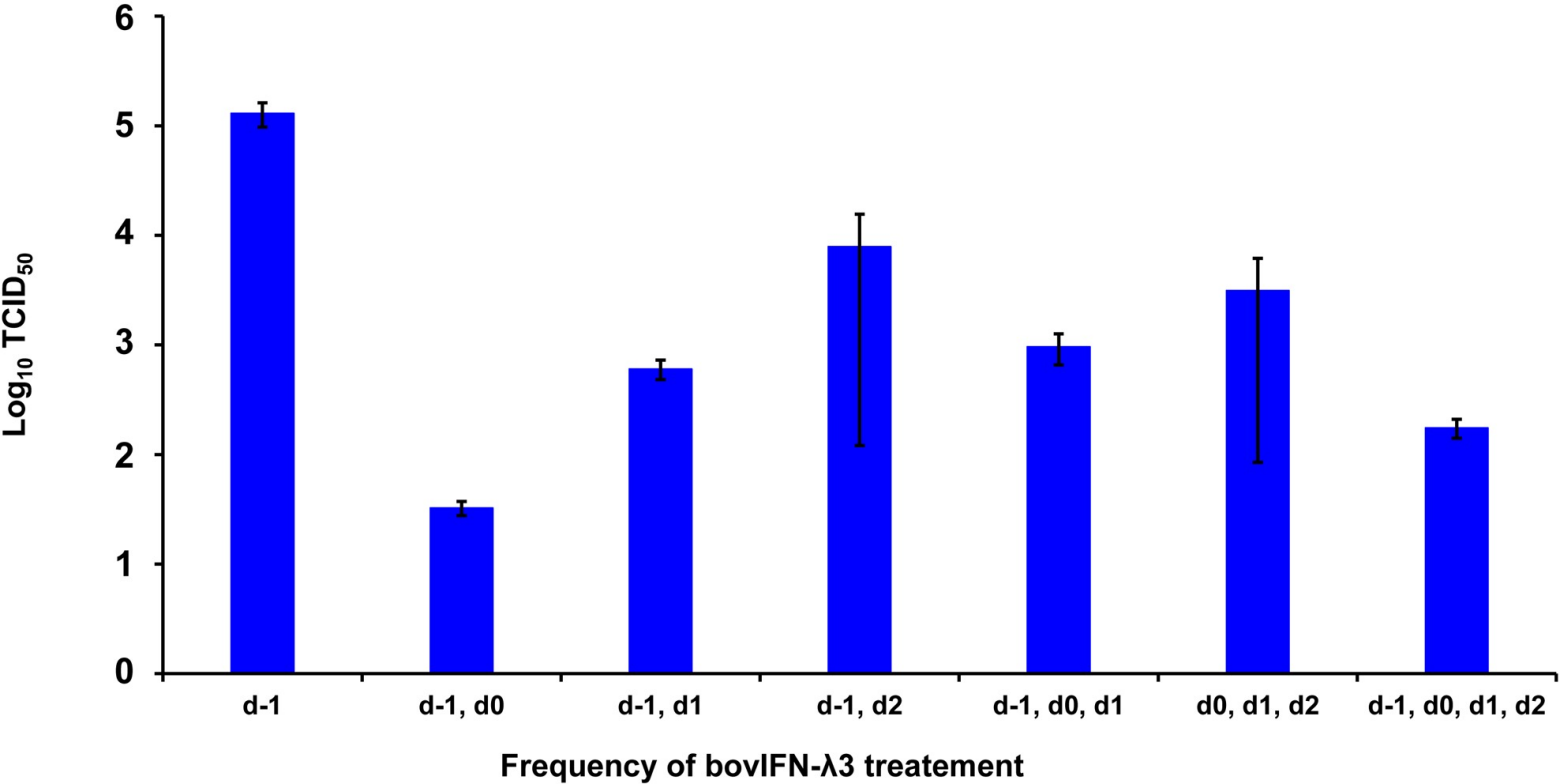
Frequency of bovIFN-λ3 treatment influenced the level of ncp BVDV inhibition in cultured Madin-Darby bovine kidney cell line (MDBK). MDBK cells were preincubated with bovIFN-λ3 (1:4 dilution) 24 hours before BVDV infection (day -1) followed by two to three additional days of bovIFN-λ3 treatment. BVDV titers were determined by BVDV RT-qPCR (VetMAX™-Gold BVDV PI Detection Kit) assay and expressed as TCID_50_.

We next sought to identify the minimum duration (number of days) of bovIFN-λ3 treatment needed to clear the virus infection. When MDBK cells were incubated with serially diluted bovIFN-λ3 (1:4-128 dilutions) for different days schedules, all groups except day -1 only, day -1/day 2, and day 0/day 1/day 2 treatment showed the highest antiviral activity against BVDV (**Figure 4**). This activity was very similar when compared to treatment for four consecutive days (**Figures 2F**, **3**). Presumably, the lack of a dose-response effect (1:4-128 dilutions; **Figure 3**) for bovIFN-λ3 against BVDV is most likely attributed to the higher concentrations of bovIFN-λ3 present in the culture supernatant. No attempt was made to identify the minimal effective dose of bovIFN-λ3 supernatant against BVDV. Intriguingly, when the MDBK cells were infected with BVDV on the same day of bovIFN-λ3 treatment (day -1), a greater BVDV staining reduction was observed only with lower bovIFN-λ3 dilutions (1:4 and 1:8) compared to the higher dilutions (1:16-128), regardless of the treatment group combination (data not shown). Although reduced BVDV staining was observed during BVDV and bovIFN-λ3 co-incubation (on day -1), a more pronounced BVDV staining reduction was observed when the MDBK cells were treated with bovIFN-λ3 24 hours before BVDV infection (**Figure 2**). This suggests that preincubation with bovIFN-λ3 is needed to stimulate an antiviral state in MDBK cells leading to a better BVDV clearance (**Figure 4**).

### 3.2 bovIFN-λ3 does not elicit antiviral activity against BVDV in BTu cells

**Figure 5:**
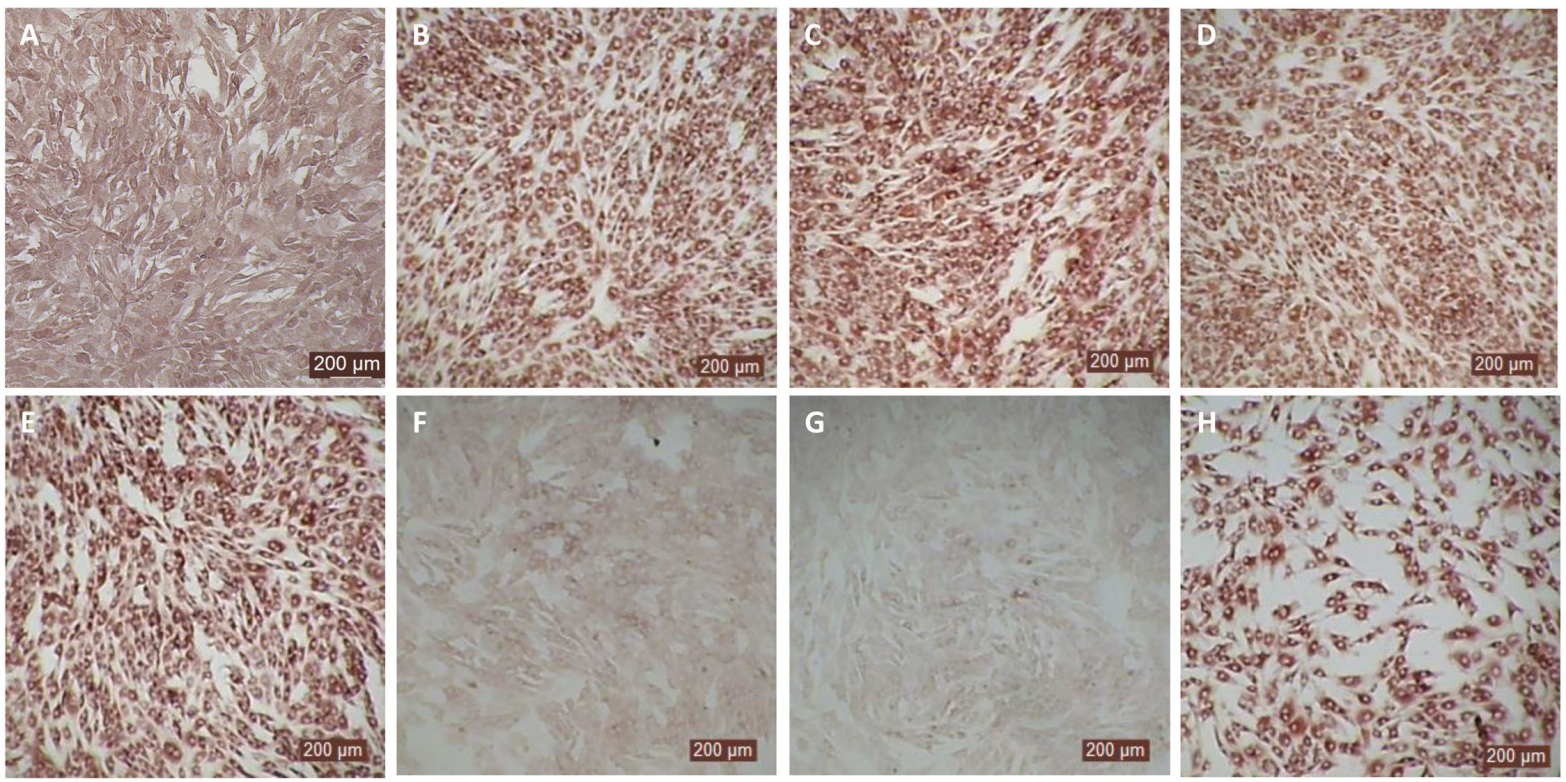
Antiviral activity bovIFN-λ3 against ncp BVDV in cultured bovine turbinate primary epithelial cells (BTu) following IL-28Rα and IL-10Rβ plasmids transfection. **(A)** Uninfected BTu cells. **(B)** BTu cells incubated with lipofectamine and BVDV (transfection control). **(C)** BTu cells infected with BVDV (no bovIFN-λ3 treatment). **(D)** BTu cells treated with bovIFN-λ3 before BVDV infection. **(E)** BTu cells transfected with IL-10Rβ expression plasmid, treated with bovIFN-λ3 before BVDV infection. **(F)** BTu cells transfected with IL-28Rα expression plasmid, treated with bovIFN-λ3 before BVDV infection. **(G)** BTu cells co-transfected with IL-28Rα and IL-10Rβ expression plasmids, treated with bovIFN-λ3 before BVDV infection. **(H)** BTu cells transfected with Mx1 expression plasmid, treated with bovIFN-λ3 before BVDV infection. In all the experiments, (**D-H**), BTu cells were treated with bovIFN-λ3 (1:16 dilution) 24 hours before BVDV infection (BVDV-2a, PI28 strain at MOI of 0.5) followed by three additional days of bovIFN-λ3 treatment (day 0, day 1 and day 2). BVDV was identified by immunoperoxidase staining using anti-E2 protein specific monoclonal N2 antibody. BVDV staining (red/brown) is restricted to the cytoplasm.

Since the nasal passage is the typical site of entry for BVDV during acute infection, next we studied whether bovIFN-λ3 could inhibit BVDV replication in primary bovine turbinate (BTu) epithelial cells, which are more relevant to the actual route of infection. We initially trialed bovIFN-λ3 on a single BTu preparation (9/08) of relatively low passage number (under 10), in a manner similar to the MDBK cell experiments. BTu cells were treated with bovIFN-λ3 one time at day -1 (or 24 hours before BVDV infection) with 2-4 days of treatment. To our surprise, BTu cells treated with bovIFN-λ3 did not demonstrate any reduction in BVDV staining at any of the bovIFN-λ3 concentrations (1:4-128) tested even with the multiple days (up to four days) of treatment (**Figure 5D**). RT-qPCR analysis revealed the similar BVDV titers present across bovIFN-λ3 dilutions (TCID_50_ = ~5 × 10^5^ - 9 × 10^5^) (**Figure 3**). The levels of BVDV staining in bovIFN-λ3 treated cells were very similar to BVDV infected control cells (**Figures 5C, D**). To verify whether this observation was specific to this particular BTu preparation, we tested four additional BTu preparations (ATCC-1390, -10/07 -12/14, and BoTur) along with BTu-9/08 (as a control). All five BTu preparations failed to demonstrate any reduction in BVDV staining suggesting BTu cells are defective in responding to bovIFN-λ3. MDBK cells were used as an assay control, and as expected, BVDV staining, and titers were greatly reduced after 2-4 days of bovIFN-λ3 treatment (**Figures 2F**, **3**).

### 3.3 Transient transfection of IL-28Rα restored antiviral activity of bovIFN-λ3 against BVDV in BTu cells

In human and mice, expression of IL-28Rα is largely restricted to epithelial cells, and the response to IFN-λ correlates with IL-28Rα expression (38, 41, 62). Unfortunately, we could not assess the IL-28Rα protein expression in BTu and MDBK cells due to the unavailability of a bovine IL-28Rα-specific antibody. To evaluate if the reduced or absence of sensitivity to bovIFN-λ3 in BTu cells was due to a decreased expression of type III IFNs receptor, both receptor subunit (IL-28Rα and IL-10Rβ) were cloned into mammalian expression plasmid vectors. Two hours after plasmid transfection cells were treated with bovIFN-λ3 (1:16 dilution, four consecutive days) followed by infection with BVDV. Over 90% of BVDV positive staining was detected in cells treated with lipofectamine alone, lipofectamine/bovIFN-λ3 and lipofectamine/pIL-10Rβ, (**Figures 5B-E**). Interestingly, BTu cells transfected with lipofectamine/IL-28Rα alone (**Figure 5F**) or co-transfected with lipofectamine/IL-28Rα/IL-10Rβ (**Figure 5G**) followed by bovIFN-λ3 treatment clearly showed a decrease in BVDV staining with little positive signal (**Figures 5F, G**). The reduced BVDV staining observed with BTu cells that IL-28Rα or IL-28Rα/IL-10Rβ overexpression (**Figures 5F/G**) was similar to that obtained for MDBK cells treated with bovIFN-λ3 (**Figure 2F**). Mx1, a GTPase, is an ISG which plays a vital role in reducing influenza A and other viral infections in mice (63). However, BTu cells transfected with the Mx1 expressing plasmid and treated with bovIFN-λ3 did not show clear reduction in BVDV staining (**Figure 5H**). Transient expression of IL-28Rα in transfected BTu cells was confirmed by flow cytometry using anti-FLAG tag specific mAb (**Supplementary Figure 1**).

### 3.4 bovIFN-α elicit antiviral activity against BVDV in BTu cells

**Figure 6:**
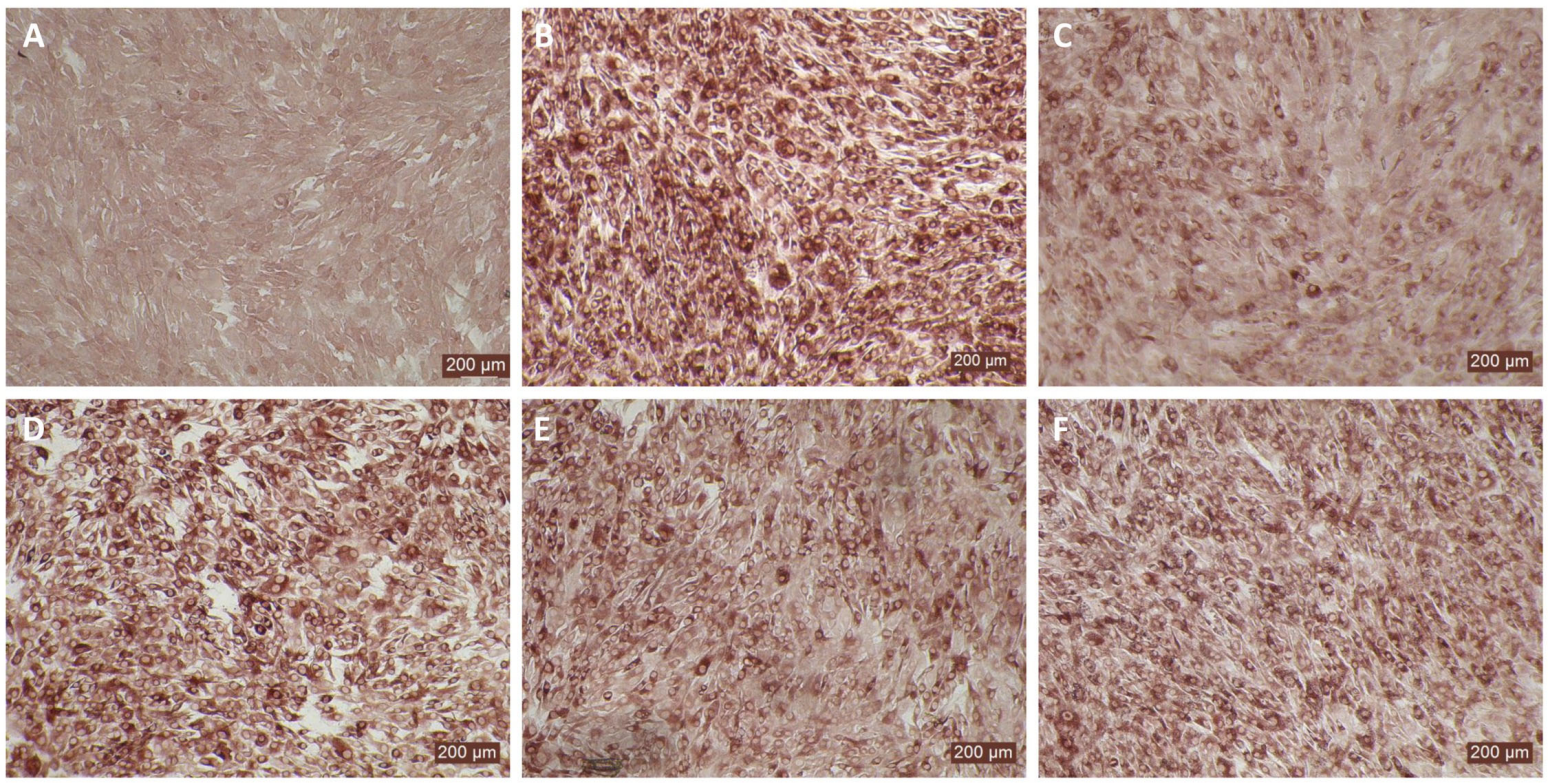
Antiviral activity of bovIFN-α against ncp BVDV in cultured bovine turbinate primary epithelial cells (BTu). **(A)** Uninfected BTu cells. **(B)** BVDV infected BTu cells. **(C)** BTu cells treated with bovIFN-α (100 ng/mL) 24 hours before BVDV infection (day -1) followed by three additional days of bovIFN-αtreatment (days 0, 1 and 2). **(D)** BTu cells treated with bovIFN-α (100 ng/mL) 24 hours before BVDV infection (day -1) followed by two additional days (day 0 and day 1) of bovIFN-α treatment. **(E)** BTu cells treated with bovIFN-α (100 ng/mL) 24 hours before BVDV infection (day -1) followed by two additional days (day 0 and day 2) of bovIFN-α treatment. **(F)** BTu cells treated with bovIFN-α (100 ng/mL) 24 hours before BVDV infection (day -1) followed by one additional day (day 1) of bovIFN-α treatment. ncp BVDV2a PI28 strain at MOI of 0.5 was used to infect BTu cells. BVDV was identified by immunoperoxidase staining using anti-E2 protein specific monoclonal N2 antibody. BVDV staining (red/brown) is restricted to the cytoplasm.

MDBK and BTu cells are commonly used for BVDV propagation, titration, and neutralization assays because both cell types show similar sensitivity to BVDV infection. Although BTu cells failed to elicit antiviral activity against BVDV following bovIFN-λ3 treatment (**Figures 3**, **5D**), it has been previously reported that BTu cells were sensitive to type I IFN treatment (IFN-α) and displayed antiviral activity against BVDV (24). We sought to confirm the type I IFN sensitivity of BTu cells using bovIFN-α under our experimental conditions. BTu cells treated four consecutive days with bovIFN-α demonstrated a greater BVDV reduction especially at higher concentrations (50-100 ng/mL) as determined by reduced BVDV staining (~20% BVDV positive cells, **Figure 6C**). When BTu cells were treated two to three days with bovIFN-α, only a partial BVDV clearance was observed even at higher bovIFN-α concentrations (50-100 ng/mL; **Figures 6D-F**). BTu cells in control wells showed no BVDV staining (**Figure 6A**) whereas BVDV infected control wells showed >90% BVDV positive cells (**Figure 6B**).

### 3.5 BTu and MDBK cells express bovIFN-λ3 receptor (IL-28Rα) with intact transmembrane domain

It has been previously reported that human PBMCs (16) and several swine cell types (54) expressed two-to-three alternative spliced forms of the IL-28Rα subunit (64). Perez et al., 2014 reported that swine IB-RS2 cells were non-responsive to swine IFN-λ3 treatment, and the lack of antiviral activity was attributed to the expression of an IL-28Rα subunit without a transmembrane domain (54). To determine whether BTu cells also express IL-28Rα variant similar to IB-RS2 cells, primers covering the transmembrane domain (exons 5 and 6) were designed and a PCR assay was performed. We observed an expected band (and several non-specific extra bands) following PCR amplification (data not shown). DNA sequencing analysis revealed the presence of an intact transmembrane domain (WALLLLLPFLLPLLLAVAIGPVMW, **Supplementary Figure 2**). No sequences lacking a transmembrane domain were observed, suggesting BTu cells lack two-to-three alternative spliced forms of the IL-28Rα subunit. Although the full-length IL-28Rα gene was amplified from MDBK cells using UTR-specific primers (one PCR product), we could not amplify the full-length IL-28Rα gene from BTu cells using the same UTR and CDS-specific primers in multiple attempts under different assay conditions. However, we were able to confirm the expression of IL-28Rα mRNA in BTu cells by using primers that targeted multiple exons such as, exons 2-3, 5-6 (transmembrane region) in addition to exon 7. We did not find any single nucleotide polymorphisms (SNPs) from the amplified exons of BTu IL-28Rα subunit.

### 3.6 Gene expression analysis of type I and III IFN signaling pathways revealed that differences exist between MDBK and BTu cells

**Figure 7:**
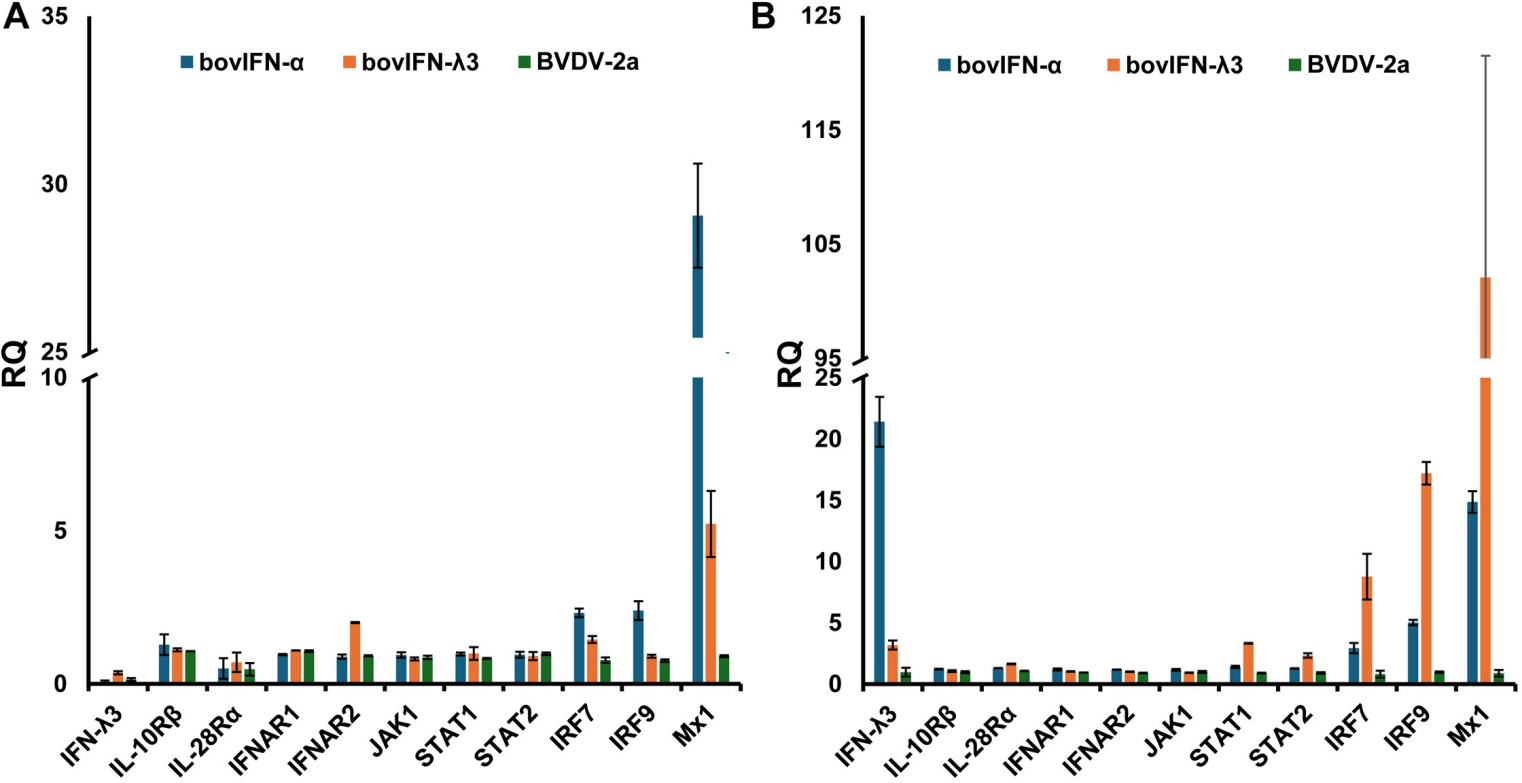
Type I and type III IFN pathway gene expression profiles in bovine turbinate primary epithelial cells (BTu) and Madin-Darby bovine kidney cell line (MDBK). BTu **(A)** and MDBK **(B)** cells were incubated with bovIFN-α (100 ng/mL), bovIFN-λ3 (1:16 dilution), or bovine viral diarrhea virus (BVDV-2a, PI28 strain at MOI of 0.5) for 24 hours. Total cellular RNA was extracted, and one-step *Taq*man RT-qPCR assay was performed. Relative quantities (RQ) of type I and III IFN signaling pathway genes, IFN receptors, and interferon stimulated genes (Mx1) were calculated using 2(−ΔΔCt) method.

We utilized a RT-qPCR assay to investigate gene expression levels in the JAK-STAT pathway, IRFs, IFN receptors, and the IFN-inducible GTPase Mx1. Relative quantities (RQ) of genes of interests (GOI) were calculated using 2^(-ΔΔCt)^ method. MDBK and BTu cells infected with BVDV demonstrated no changes in the tested genes expression levels suggesting BVDV did not interfere with the type IFN I and III signaling pathway (**Figure 7**). There were no changes in either cell respective receptor gene expression type treated with type I or III IFNs except that BTu cells showed an ~2-fold increase of IFNAR2 expression in response to bovIFN-λ3 treatment (**Figure 7**). Interestingly, ~2-3-fold increase in STAT1 and STAT2 expression was observed in MDBK cells following bovIFN-λ3 treatment (**Figure 7**). Increased expression of IRF7 (~3-8-fold) and IRF9 (~2-6-fold) was observed in both cell types following bovIFN-α treatment (**Figure 7**). MDBK cells exhibited a 9-fold increase in IRF7 expression and a 17-fold increase in IRF9 expression following bovIFN-λ3 treatment, while only a 3-fold increase in IRF7 expression and a 5-fold increase in IRF9 expression was observed in BTu cells following bovIFN-α treatment (**Figure 7**). MDBK cells treated with bovIFN-λ3 showed a 102-fold increase in Mx1 gene expression compared to a 15-fold increase with bovIFN-α. As expected, BTu cells were found to have higher Mx1 gene expression (~29-fold) in response to bovIFN-α treatment compared to bovIFN-λ3 (~5-fold) treatment. MDBK cells showed an unexpected increase in IFN-λ3 gene expression (21-fold) when treated with bovIFN-α, compared to a modest IFN-λ3 gene expression (~3-fold) when the cells were treated with bovIFN-λ3. It is important to highlight that multiple cell types (epithelial, myeloid, and lymphoid cell lines) including nasal turbinate cells (derived from ferrets), produce IFN-λ following various virus infection (15, 16, 65, 66). However, ncp BVDV-2a (PI28) did not have any effect on IFN-λ3 expression in either BTu or MDBK cells (**Figure 7**).

### 3.7 Transcriptomic analyses revealed reduced IL-28Rα (IFNLR1) transcripts expression in BTu cells

**Figure 8:**
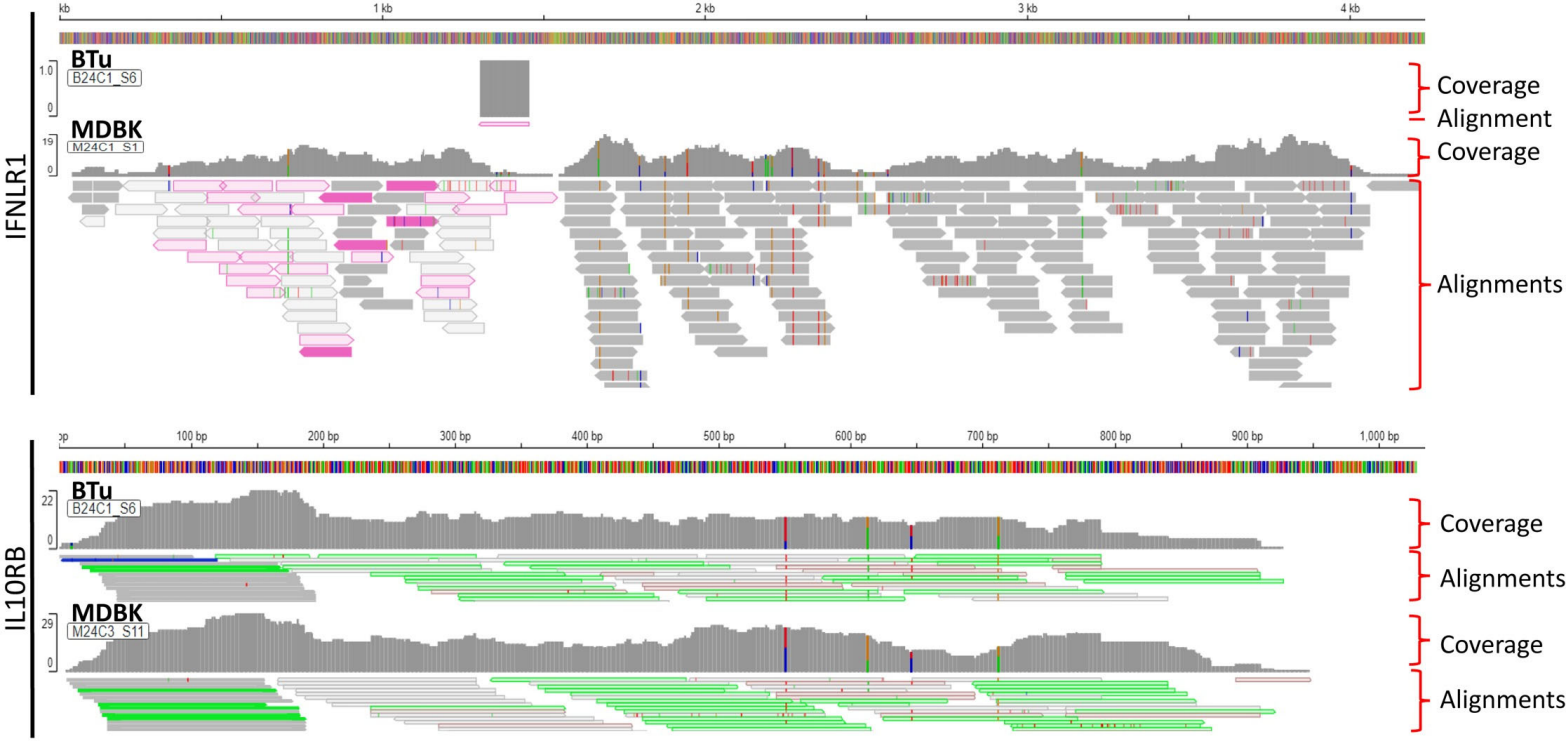
Visualization of Type III interferon (IFN) receptors (IFNLR1 and IL10RB) from RNA-Seq data obtained from bovine turbinate (BTu) and Madin-Darby bovine Kidney (MDBK) cells. Each panel includes total coverage and transcripts read alignments. Integrative Genomics Viewer was used to visualize the transcripts and coverage.

Since we could not amplify full-length IL-28Rα (IFNLR1) DNA sequence by PCR, but RT-qPCR analyses suggested thar there was a lower IFNLR1 expression in BTu cells as compared to MDBK cells, transcriptomic libraries from BTu cells were prepared and analyzed to have broader perspective of gene expression. MDBK cells were used as a control. RNA sequencing indicated the differentially expressed transcripts were present in both cell types (**Table 2**). Both cell types had two-to-three transcripts for IRF7, IRF9, STAT1, Mx1, and IL10RB and both cell types exhibited similar expression preferences for the given transcript (**Table 2**). Approximately two-fold higher JAK1 and STAT1 transcript reads were found in BTu cells as compared to MDBK cells while both cell types had similar transcript reads for IRF3, IRF9, IFNAR1 and IFNAR2. Interestingly, only one RNA fragment for IFNLR1 out of three libraries was found in BTu cells, whereas MDBK cells had multiple transcripts to cover the entire IFNLR1 sequence (**Figure 8**). This observation was similar to RT-qPCR finding and confirms the lack of IFNLR1 expression in BTu cells as compared to MDBK cells. Unlike type I IFN receptors (IFNAR1 and IFNAR2), two-to-three fold higher IL10RB transcript reads were also found in MDBK cells compared to BTu cells (**Table 2**; **Figure 8**). As expected, uninduced (control) cells did not express IFN-λ3 transcripts.

**Table 2:** Summary of transcript abundance in uninduced bovine turbinate cells (BTu) and Madin-Darby bovine kidney cell (MDBK) cells.

Gene	Transcript ID	BTu (TPM)*	MDBK (TPM)*	P-value	FDR
**JAK1**	ENSBTAT00000004101	54.6 ± 19.1	28.6 ± 3.2	5.5728E-05	3.0095E-04
**STAT1**	ENSBTAT00000061553	0.0	0.5 ± 0.9	2.2636E-02	5.5609E-02
**STAT1**	ENSBTAT00000084342	1.0 ± 1.4	0.0	1.4668E-03	5.4655E-03
**STAT1**	ENSBTAT00000010351	56.4 ± 26.8	27.3 ± 3.8	1.0724E-04	5.4327E-04
**STAT2**	ENSBTAT00000005749	12.2 ± 6	9.4 ± 1.2	4.0886E-01	5.6202E-01
**IRF3**	ENSBTAT00000031869	51.4 ± 11.9	52.8 ± 6.8	6.2860E-18	1.3708E-16
**IRF7**	ENSBTAT00000065006	2.1 ± 0.4	22.6 ± 5.6	8.5046E-07	6.4204E-06
**IRF7**	ENSBTAT00000075628	0.0	3.6 ± 2.6	7.5573E-01	8.5061E-01
**IRF9**	ENSBTAT00000064992	1.8 ± 1	1.4 ± 0.2	5.5859E-01	6.9810E-01
**IRF9**	ENSBTAT00000036455	2.6 ± 1.5	1.7 ± 0.5		
**IFNL3**	ENSBTAT00000068972	0.0	0.0	2.5557E-06	1.7810E-05
**Mx1**	ENSBTAT00000012035	0.0	2.2 ± 0.9		
**Mx1**	ENSBTAT00000071501	0.0	0.1 ± 0.2		
**Mx1**	ENSBTAT00000043742	0.0	0.1 ± 0.2	4.4796E-12	6.3121E-11
**GAPDH**	ENSBTAT00000037753	2346.4 ± 750.7	4331.6 ± 453.8	6.5251E-04	2.6971E-03
**GAPDH**	ENSBTAT00000078656	19.1 ± 7.8	36.9 ± 10.9	3.1108E-04	1.4025E-03
**GAPDH**	ENSBTAT00000067004	0.8 ± 0.8	7.2 ± 0.8	7.2926E-01	8.3266E-01
**IFNAR1**	ENSBTAT00000029080	26.3 ± 11.5	25.7 ± 3.2	8.1496E-01	8.9132E-01
**IFNAR2**	ENSBTAT00000020239	6.3 ± 3.1	6.2 ± 0.5	3.7292E-02	8.3357E-02
**IL10RB**	ENSBTAT00000073488	6.6 ± 1.7	13.9 ± 2.3	2.9129E-04	1.3253E-03
**IL10RB**	ENSBTAT00000025852	4.0 ± 1.7	11.5 ± 1.6		
**IL10RB**	ENSBTAT00000078494	0.0	0.0	1.6575E-11	2.2273E-10
**IFNLR1**	ENSBTAT00000001456	0.0	3.4 ± 0.6	6.5383E-01	7.7565E-01

TPM, Transcripts Per Million, a relative measure of transcript abundance. *TPM is shown in means ± SD. FDR, False discovery rate, BTu and MDBK cells were seeded into 6-well plates and total cellular RNA was extract 24 hours later. Transcriptomic libraries were prepared and sequenced using Illumina NovaSeq 6000 Sequencing System.

## 4 Discussion

A new class of IFNs, type III IFNs or IFN-λ were independently identified in 2003 by two groups looking for new cytokines of the IL-10 family (15, 16). Indeed, the type III IFN family receptor complex consists of the widely expressed IL-10Rβ subunit and a unique IL-28Rα (IFNLR1) subunit (15, 16, 20). Later studies suggested that the type III IFN response is largely restricted to epithelial cells of the gastrointestinal and reproductive tracts, kidney, brain, and liver due to the specific expression of the IL-28Rα subunit in those tissues, and it is unique to IFN-λ signaling (38, 41, 62). One member of the type III IFN family, IFN-λ3/IL-28B, was identified in primary embryonic bovine kidney cells, and its antiviral activity was confirmed against several viruses both *in vitro* in cell culture and *in vivo* in animal studies (31, 42, 43). Many studies have used the MDBK cell line to demonstrate antiviral activity of bovIFN-λ3. Since the primary site of BVDV entry into cattle is oronasal route during acute infection and first replicates virus in nasal mucosa (44, 45), the aim of this study was to assess bovIFN-λ3 antiviral activity in primary bovine turbinate cells (BTu), a more relevant BVDV susceptible epithelial cell type.

The first cellular response and defense mechanism against viral infections in vertebrates is the expression of type I (IFN-α/β) and type III (IFN-λs) IFNs (14–16). Despite binding to different receptors, type I and III IFNs produce cellular antiviral responses that are similar and utilize the same JAK-STAT signaling pathway and induce the expression of hundreds of ISGs including Mx1 (15, 18, 19). Although most viruses induce a type I and type III response, ncp BVDV (but not cp BVDV) is known to inhibit *in vitro* type I IFN response in cell culture (5, 21–24). This inhibition has been attributed to the prevention of binding of transcription factor IRF3 to nuclear DNA (25). Later studies revealed that the N^Pro^ protein of BVDV was responsible for IRF3 binding inhibition (67). Similarly, we did not observe any upregulation of tested genes such as type I and III IFNs receptors, JAK-STATs, IRFs, and Mx1, further confirming the immunosuppression activity of ncp BVDV in both MDBK and BTu cells (**Figure 7**). Treatment of MDBK and bovine white blood cells with IFN-α, as well as infection with bovine herpes virus 1, and bovine rotavirus upregulated Mx1 gene expression whereas, infection of bovine endometrial cells with ncp BVDV (Pe515nc strain) reduced/inhibited Mx1 and Mx2 gene expression (68, 69). Furthermore, the ncp BVDV strain used in this study (PI28 strain) did not induce either the up regulation of Mx1 gene expression in both MDBK and BTu cells (**Figure 7**). As it has been previously reported, higher Mx1 (transcripts) expression was observed with bovIFN-λ3 (~102-fold) and bovIFN-α (~15-fold) treated MDBK cells (31, 68) (**Figure 7**). Higher Mx1 expression was measured in BTu cells following treatment with bovIFN-α (~29-fold), but lower induction was observed with bovIFN-λ3 (~5-fold) treatment (**Figure 7**). Since both MDBK and BTu cells responded well to bovIFN-α treatment with upregulation of Mx1 gene expression, these findings suggest JAK-STAT signaling pathway is intact in both cell types. However, observed modest up regulation of Mx1 gene expression in BTu cells compared to MDBK cells during bovIFN-λ3 treatment may indicate that BTu cells’ sensitivity was lower to IFN-λ3. It is important to highlight that only a few Mx1 transcripts were found in uninduced and uninfected cells, either MDBK or BTu, while two-fold higher JAK1, STAT1, and IRF9 transcript numbers were found in BTu cells as compared to MDBK cells (**Table 2**).

The Myxovirus resistance (Mx) proteins are evolutionary conserved in vertebrates and show dynamin-like GTPases activity and produced due to the activation of type I and III IFNs following viral infections. Mx gene expression is tightly controlled and their expression is induced only by type I and III IFNs and not directly by viruses (63). It is well-known that mice carrying the functional Mx1 gene inhibits influenza A viral transcription and replication, rendering them resistant to infection (70). Although ncp BVDV infection leads to the inhibition of Mx1 expression *in vitro* in cell culture, calves infected with ncp BVDV showed strong Mx1 expression (68, 69). Transfection of BTu cells with the Mx1 gene in this study failed to reduce BVDV infection (**Figure 2**). Since Mx1 inhibits influenza A viral transcription/replication at the nuclear level, the lack of Mx1 effect on BVDV may be due to BVDV replication is restricted to the cytoplasm (**Figures 1**, **2**, **5**, and **6**, staining is restricted to the cytoplasm). This finding suggests that Mx1 is unable to control BVDV infection in cell cultures.

On the other hand, African green monkey kidney (Vero) cells cannot produce IFN-β following viral infection, but can respond to IFN-β and IFN-λ treatment due to the presence of intact type I and III IFNs receptors as well as intact JAK-STAT signal pathway (71). It has also been previously reported that parainfluenza virus 3 blocks IFN-λ mediated ISGs expression in Vero cells by reducing phosphorylation of STAT1 and STAT2 (72). Since transfection of the IL-28Rα plasmid improved antiviral activity of BTu cells against BVDV (**Figure 5**), we do not expect interference of STAT1/STAT2 phosphorylation by BVDV. This conclusion is further supported by the clearance of BVDV in MDBK cells following bovIFN-λ3 as well as bovIFN-α treatments (**Figure 2**), BTu cells following bovIFN-α treatment (**Figure 6**), and finally BTu cells transfected with IL-28Rα plasmid followed by bovIFN-λ3 treatment (**Figure 5**).

When bovIFN-λ3 pretreated BTu cells were infected with BVDV followed by three consecutive days of bovIFN-λ3 treatment, we did not observe any reduction in titers (**Figure 3**) or BVDV staining (**Figure 5**). We detected this same phenotype of an absent antiviral response against BVDV in five different BTu preparations treated with bovIFN-λ3, suggesting the defect is universal (to BTu cells). However, similarly treated MDBK cells, whether incubated with bovIFN-λ3 for one or four consecutive days, showed increased antiviral activity against BVDV (**Figures 3**, **4**). Since the JAK-STAT signaling pathway is intact in BTu cells as indicated by the positive response following bovIFN-α treatment (**Figure 7**) as well as reduced BVDV staining (**Figure 6**), these findings prompted us to suspect a defect in type III IFNs receptors (IL-28Rα and/or IL-10Rβ) expression. However, our RT-qPCR assays with both MDBK and BTu cells showed basal expression levels of both receptors after normalization to control samples (**Figure 7**). Differential splicing of IL-28Rα gene of humans produce three different mRNAs. The first variant leads to the expression of the functional receptor; the second lacks a part of the intracellular domain and is nonfunctional; and the third encodes only the extracellular part of the receptor lacking transmembrane domain (16, 64). Similar to the BTu cells in this study, the lack of response of porcine primary IB-RS2 cells to porcine IFN-λ3 treatment has also been reported (54). However, PCR amplification followed by sequencing of porcine IL-28Rα revealed the expression of IL-28Rα receptor without a transmembrane domain (54). Since we targeted exon 7 region of IL-28Rα for RT-qPCR assays, we designed new primers targeting exons 5-6 (transmembrane region) and exons 2-3. Both RT-qPCR primers worked in MDBK and BTu cells and no difference was observed in their respective expression levels, confirming the expression of IL-28Rα subunit. Next, we sequenced the exons 5-6 PCR products and found the sequence contained the intact transmembrane domain (**Supplementary Figure 2**). This finding suggests that the lack of IL-28Rα splicing variants in BTu cells. Simar to RT-qPCR findings, transcriptomic analyses also revealed the lack of IL-28Rα (IFNLR1) transcripts expression in BTu cells as compared to MDBK cells (**Table 2**; **Figure 8**).

In addition to the expression of IL-28Rα splice variants in humans, it has been reported that differential expression of IL-28Rα can also determine the magnitude of antiviral response (73). We consistently observed lower Ct values (~20-25) for IL-28Rα transcripts with MDBK cells compared to relatively higher Ct values (~30-36) in BTu cells, irrespective of which exon was targeted. These findings suggests that there is a reduced expression of IL-28Rα transcripts and consequently a reduction in IL-28Rα expression of BTu cells compared to MDBK cells. However, we could not determine the IL-28Rα expression in BTu or MDBK cells due to the lack of availability of bovine IL-28Rα subunit specific antibody. It has been previously reported the IL-10Rβ subunit is widely expressed in many cell types (15, 16). Consistently, we observed relatively low Ct values of IL-10Rβ in both MDBK and BTu cell types (~20-21) suggesting similar expression levels of IL-10Rβ subunits between both cell types. Our transcriptomic analyses confirmed IL-10Rβ (IL10RB) expression in both cell types although MDBK cells showed approximately two-fold higher IL10RB transcript reads compared to BTu cells (**Table 2**; **Figure 8**).

We successfully amplified the IL-28Rα CDS from RNA extracted from MDBK cells; however, we could not amplify IL-28Rα CDS from RNA extracted from BTu cells despite the use of various primer sets and optimal reaction conditions. In order to circumvent this difficulty, transcriptomic libraries were prepared and sequenced. Unfortunately, we could not determine the IL-28Rα sequence since no IL-28Rα transcripts were found in BTu cells (**Figure 8**). There have been multiple SNPs identified in the human IL-28Rα gene and some of the SNPs were associated with the severity of allergic rhinitis (74). We could not find any such SNPs in the limited regions of the IL-28Rα gene that we amplified from BTu cells. It is known that expression of IL-28Rα on epithelial cells is required for IFN-λ antiviral activity (40). Therefore, to better understand the lack of BTu cells response to bovIFN-λ3 treatment, both receptors were synthesized (gBlock gene fragments), cloned into mammalian expression plasmids, and transfected into BTu cells. Interestingly, BTu cells transfected with the IL-28Rα plasmid, but not the IL-10Rβ plasmid, showed improved antiviral activity against BVDV after bovIFN-λ3 treatment (**Figure 5**). The increased antiviral activity of the IL-28Rα plasmid transfected BTu cells was very similar to the antiviral activity observed with MDBK cells against BVDV (**Figures 2**, **3**). These findings together with transcriptomic analyses strongly indicate that BTu cells express minimal IL-28Rα subunit, which likely accounts for the lack of response to bovIFN-λ3 treatment.

Despite MDBK cells being highly responsive to bovIFN-λ3, a recent study found that porcine kidney cells (PK-15) failed to clear pseudorabies virus following porcine IFN-λ3 pretreatment (39). The lack of antiviral activity of PK-15 cells to porcine IFN-λ3 was attributed to the reduced IL-28Rα expression (39). In a manner similar to what we observed in this study with bovIFN-α treated BTu cells inactivating BVDV, porcine IFN-α treated PK-15 cells also inhibited pseudorabies virus replication. These findings suggest that some epithelial cells are not as responsive to IFN-λ3 as others, and the reduced responsiveness (to IFN-λ3) is attributed to the reduced IL-28Rα expression. It is important to highlight that although the expression of IL-28Rα is indicative of responsiveness to IFN-λ in epithelial cells, secretory variant of IL-28Rα in leukocytes can bind and abolish IFN-λ effects despite leukocytes express similar levels of IL-28Rα on the cell surface (75).

## 5 Conclusions

In conclusion, we used RT-qPCR, RNA-Seq (transcriptome), cell culture, and IFN-λ3 receptor plasmid transfection to characterize BTu cell response to bovIFN-λ3 treatment. We did not identify any IL-28Rα alternative spliced variants or SNPs in the regions amplified by PCR from the BTu cells. However, RT-qPCR findings indicated that there was a reduced expression of IL-28Rα transcripts in BTu cells as compared to MDBK cells. Transcriptomic analyses also revealed the lack of IL-28Rα transcripts expression in BTu cells. Since transfection of BTu cells with a plasmid encoding the IL-28Rα subunit established bovIFN-λ3 mediated antiviral activity against BVDV, reduced IL-28Rα subunit expression in BTu cells is most likely to be the cause for the lack of response to bovIFN-λ3 treatment. Regardless, our results indicate that the levels of the IL-28Rα subunit are crucial to effectively transduce the antiviral activity induced by bovIFN-λ3.

## Data Availability

The data presented in the study are deposited in the NCBI SRA repository, accession number PRJNA1124102.

## Supplementary material

Supplementary Figure 1 Transfected bovine turbinate primary epithelial cells (BTu) expressed bovine IL-28Rα on the cell surfaces. BTu cells were transfected with a mammalian expression plasmid containing FLAG epitope inserted between signal sequence and extracellular domain of bovine IL-28Rα. Parent un-transfected and IL-28Rα transfected BTu cells were incubated with anti-FLAG M2 primary mAb (IgG1) followed by incubation with Alexa Fluor 488 anti-mouse IgG1 mAb. Transient expression of bovine IL-28Rα on the cell surfaces was analyzed by flow cytometry.

Supplementary Figure 2 IL-28Rα transmembrane domain was intact in bovine turbinate primary epithelial cells (BTu) cells. IL-28Rα transmembrane domain was amplified by PCR using exon 5 and exon 6 specific primers using cDNA prepared from BTu cells and sequenced by Sanger sequencing. Nucleotide sequences (IL28Ra-ExF5 (1) and IL28Ra-Ex5F (2)) encoding transmembrane domain (WALLLLLPFLLPLLLAVAIGPVMW; IL28Ra-TM) are shown. Sequenced were aligned using Sequencher 5.2.4 program.
