# Supplementary figures and images for: Antiviral activity of bovine type III interferon against bovine viral diarrhea virus is greatly reduced in bovine turbinate cells due to limited expression of IFN lambda receptor 1 (IL-28Rα)
Source: Front Immunol. 2024 Aug 19;15:1441908. doi: 10.3389/fimmu.2024.1441908 (PMC11366575; doi:10.3389/fimmu.2024.1441908)

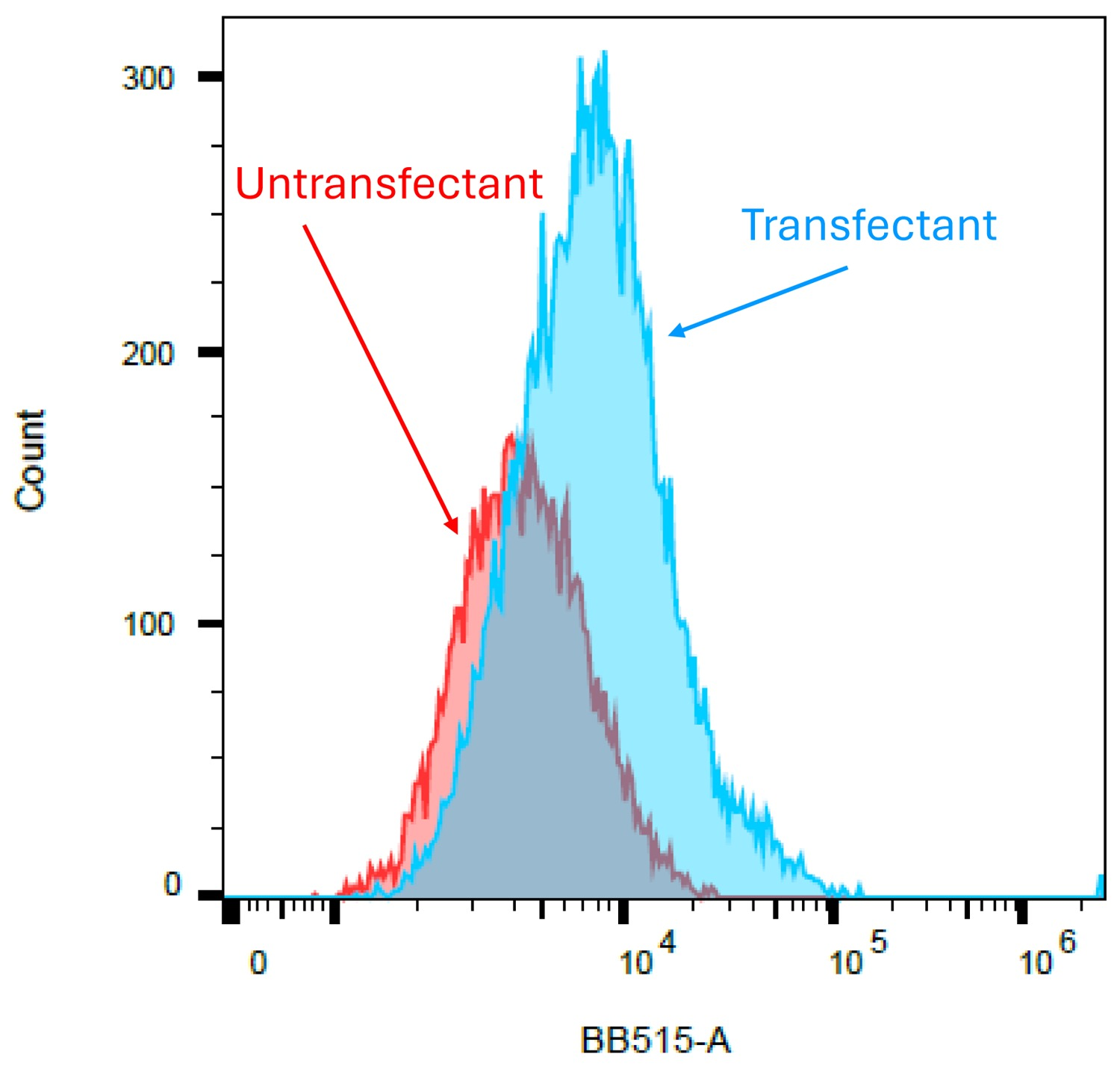

Supplement: Supplementary Figure 1 — Transfected bovine turbinate primary epithelial cells (BTu) expressed bovine IL-28Rα on the cell surfaces. BTu cells were transfected with a mammalian expression plasmid containing FLAG epitope inserted between signal sequence and extracellular domain of bovine IL-28Rα. Parent un-transfected and IL-28Rα transfected BTu cells were incubated with anti-FLAG M2 primary mAb (IgG1) followed by incubation with Alexa Fluor 488 anti-mouse IgG1 mAb. Transient expression of bovine IL-28Rα on the cell surfaces was analyzed by flow cytometry. [file Image1.tif]

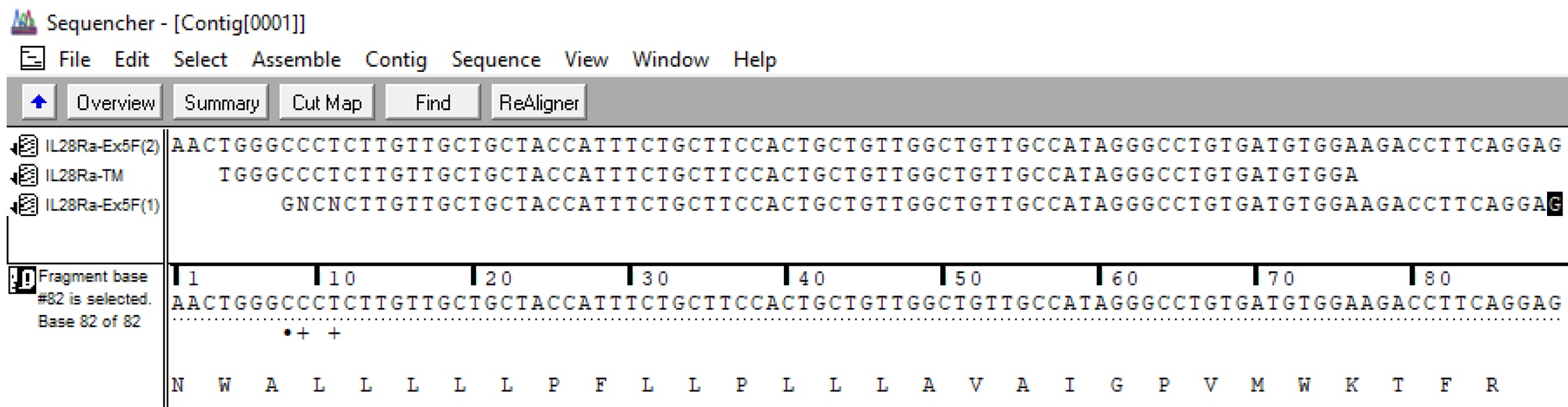

Supplement: Supplementary Figure 2 — IL-28Rα transmembrane domain was intact in bovine turbinate primary epithelial cells (BTu) cells. IL-28Rα transmembrane domain was amplified by PCR using exon 5 and exon 6 specific primers using cDNA prepared from BTu cells and sequenced by Sanger sequencing. Nucleotide sequences (IL28Ra-ExF5 (1) and IL28Ra-Ex5F (2)) encoding transmembrane domain (WALLLLLPFLLPLLLAVAIGPVMW; IL28Ra-TM) are shown. Sequenced were aligned using Sequencher 5.2.4 program. [file Image2.tif]
